# A Lgr5-independent developmental lineage is involved in mouse intestinal regeneration

**DOI:** 10.1242/dev.204654

**Published:** 2025-04-10

**Authors:** Maryam Marefati, Valeria Fernandez-Vallone, Morgane Leprovots, Gabriella Vasile, Frédérick Libert, Anne Lefort, Gilles Dinsart, Achim Weber, Jasna Jetzer, Marie-Isabelle Garcia, Gilbert Vassart

**Affiliations:** ^1^ Institute of Interdisciplinary Research in Molecular Human Biology (IRIBHM, https://iribhm.org/), Université Libre de Bruxelles (ULB), 1070 Brussels, Belgium; ^2^ Institute of Pathology and Molecular Pathology, University Hospital Zurich, University of Zurich, CH-8091 Zurich, Switzerland; ^3^ Institute of Molecular Cancer Research, University of Zurich, CH-8091 Zurich, Switzerland

**Keywords:** Intestinal development, Organoids, Regeneration, Stem cells

## Abstract

Collagenase and dispase treatment of intestinal tissue from adult mice generates cells growing in matrigel as stably replatable cystic spheroids, in addition to differentiated organoids. Contrary to classical EDTA-derived organoids, these spheroids display poor intestinal differentiation and grow independently of Rspondin, noggin and EGF. Their transcriptome strikingly resembles that of fetal intestinal spheroids, with downregulation of crypt base columnar cell (CBC) markers (Lgr5, Ascl2, Smoc2 and Olfm4). In addition, they display upregulation of inflammatory and mesenchymal genetic programs, together with robust expression of YAP target genes. Lineage tracing, cell-sorting and single cell RNA sequencing experiments demonstrate that adult spheroid-generating cells belong to a hitherto undescribed developmental lineage, independent of Lgr5-positive CBCs, and are involved in regeneration of the epithelium following CBC ablation.

## Teaser

**Summary:** Regeneration of the intestinal epithelium relies on a hierarchical stem cell model involving a previously unreported lineage of reserve stem cells in addition to the plasticity model.

## INTRODUCTION

Maintenance of the intestinal epithelium involves actively cycling Lgr5-positive (Lgr5+ve) stem cells (crypt base columnar cells, CBCs) located at the bottom of the crypts and responsible for cell renewal under homeostatic conditions (Barker et al., 2007; Beumer and Clevers, 2016). When submitted to harmful stresses causing cell death and/or affecting the integrity of the intestinal barrier, the epithelium triggers an extraordinary diversity of regenerative responses. These may involve activation of quiescent (‘+4’) stem cells (Richmond et al., 2016; Yan et al., 2012), rerouting of early secretory progenitors to stemness (van Es et al., 2012), dedifferentiation of Paneth cells (Schmitt et al., 2018; Yu et al., 2018), and enteroendocrine (Sei et al., 2018; Yan et al., 2017) or enterocyte (Tetteh et al., 2015) progenitors (for reviews, see Bankaitis et al., 2018; Capdevila et al., 2021). When activated, these various mechanisms lead to reconstitution of the Lgr5+ve stem cell pool, ready to ensure post-injury maintenance of the epithelium (Metcalfe et al., 2014). On top of this extensive phenotypic plasticity, two-way interconversion of CBCs and +4 stem cells has been demonstrated, leading to the conclusion that a hierarchical stem cell model might not apply in the intestine (Clevers and Watt, 2018; Post and Clevers, 2019; Shivdasani et al., 2021; Takeda et al., 2011; Yousefi et al., 2017).

Recent studies have demonstrated that a characteristic common to most, if not all, regenerative responses in the gut epithelium is the (re)acquisition of a fetal-like intestinal phenotype (Ayyaz et al., 2019; Fernandez et al., 2016; Nusse et al., 2018; Yui et al., 2018), with expression of part of a genetic programme first identified in fetal intestinal spheroids (Mustata et al., 2013). In all cases, the cells at the origin of the regenerating tissue were not formally identified but they were assumed to belong to the main Lgr5+ve lineage (Ayyaz et al., 2019; Yui et al., 2018).

In the present study, we show that stably replatable spheroids, strikingly resembling fetal spheroids, are generated from homeostatic adult intestine following dissociation of the tissue with collagenase and dispase (hereafter, collagenase/dispase). Lineage-tracing experiments demonstrate that the cells at the origin of these spheroids belong to a separate developmental lineage, independent of Lgr5+ve CBCs, and are involved in regeneration of the epithelium following ablation of CBCs. Our results lead to the conclusion that a hierarchical stem cell model applies to regeneration of the intestinal epithelium, in addition to the plasticity model.

## RESULTS

### Collagenase/dispase treatment of adult intestine releases spheroid-generating cells

**Fig. 1.**
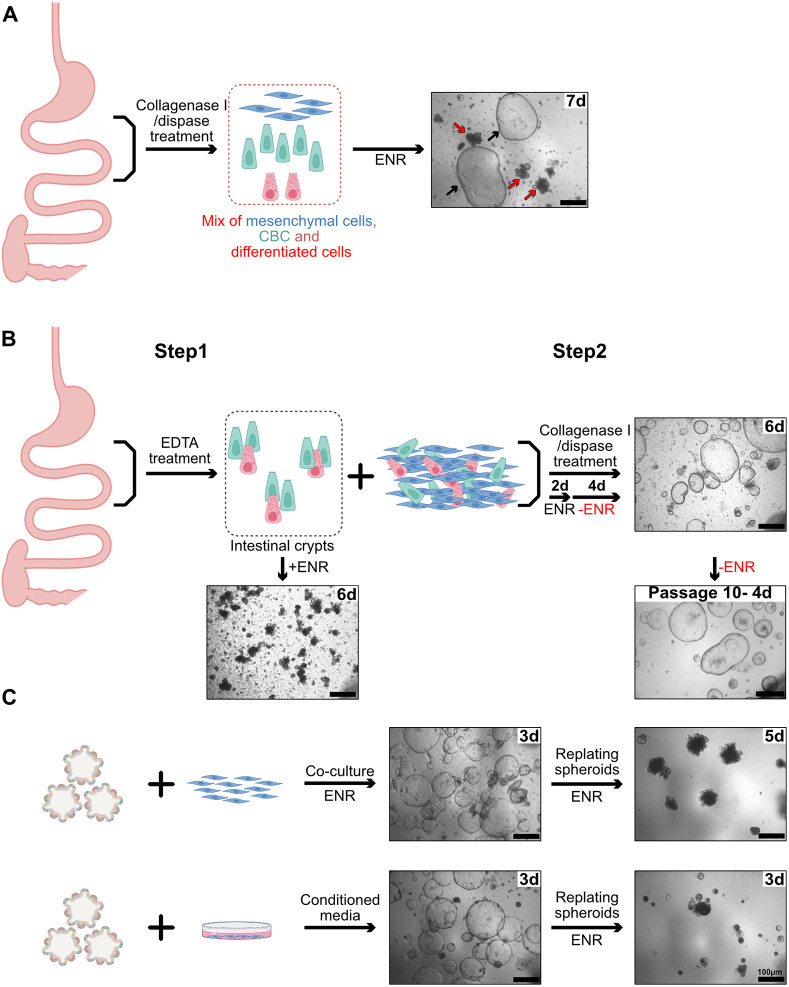
**Collagenase/dispase treatment of adult intestine releases spheroid-generating cells.** (A) Treatment of intestinal tissue with collagenase/dispase releases cellular material growing as spheroids (black arrows) when cultured in 3D, in addition to minigut organoids (red arrows). (B) EDTA treatment of intestinal tissue (step 1) releases crypts growing only as organoids, whereas action of collagenase/dispase on the material remaining after EDTA treatment (step 2) generates only spheroids that can be serially replated free of mesenchyme. (C) EDTA-derived organoids in co-culture with intestinal mesenchymal cells or in the presence of medium conditioned by intestinal mesenchyme convert to a spheroid phenotype but revert to the organoid phenotype in the absence of mesenchyme influence. Scale bars: 100 µm. Labels in the upper right corner of each picture refer to the days after initiation of cultures. Schematics created in BioRender by Hadefi, A., 2025. https://BioRender.com/v53v026. Republished with permission.

Collagenase/dispase treatment of intestinal tissue is used to isolate mesenchymal cells and grow them in 2D cultures (Powell et al., 2011). When the same protocol was used, but with the resulting material seeded in matrigel under Sato culture conditions (Sato et al., 2009), we observed growth of a mix of minigut organoids and variable numbers of cystic spherical structures resembling fetal intestinal spheroids (Fordham et al., 2013; Mustata et al., 2013) (Fig. 1A). Since the standard EDTA-based protocol (Sato et al., 2009) generates only organoids, we reasoned that the spheroids must originate from collagenase/dispase digestion of the material normally discarded after EDTA treatment, from which most of the crypts have been removed (Fig. S1A). Using a two-step protocol (see Materials and Methods, and Fig. 1B), we obtained bona fide minigut organoids from the EDTA fraction, as expected, and only spheroids, together with mesenchymal cells, from the collagenase/dispase fraction (Fig. 1B). Upon replating, these spheroids could be stably cultured free of mesenchymal cells (Fig. 1B). Morphologically similar spheroids are obtained when EDTA-derived organoids are co-cultured with mesenchymal cells or in the presence of medium conditioned by mesenchymal cells (Fig. 1C) (Lahar et al., 2011; Roulis et al., 2020)*.* Under these conditions, the switch of organoids towards spheroid morphology is in direct relation to the amount of mesenchymal cells present in the co-culture and to the factors released by them (Fig. S1B). However, contrary to collagenase/dispase-derived spheroids, spheroids derived from organoids revert to bona fide organoids as soon as they are freed from mesenchymal cells, or upon withdrawal of the conditioned medium (Fig. 1C). Using the classical matrigel dome-embedding protocol (but see below), the yield of adult collagenase/dispase-derived spheroids was variable, with, on average, one out of three mice being productive.

### Adult intestinal spheroids grow in absence of Rspondin, EGF and noggin

**Fig. 2.**
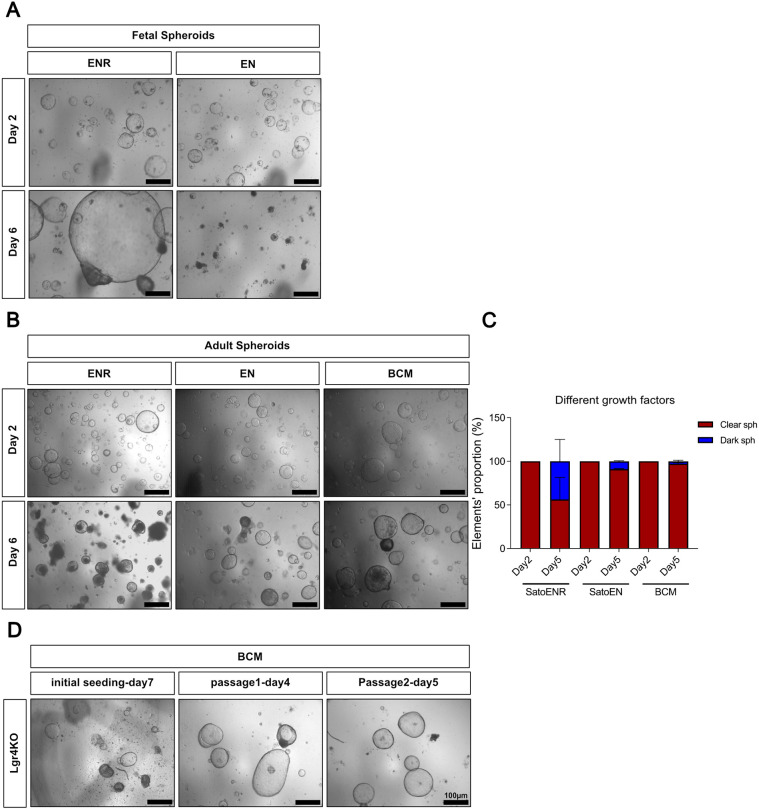
**Adult intestinal spheroids grow in the absence of EGF, noggin and Rspondin.** (A) Fetal spheroids derived from E15.5 embryonic intestine rely on Rspondin for their growth *in vitro* (ENR versus EN). (B) Adult spheroids can be cultured in the presence of EGF, noggin and Rspondin (ENR) medium as well as of EN medium, but thrive better in plain medium devoid of growth factors (BCM). (C) The proportion of dark and clear spheroids at day 2 and day 6 after being in different culture media. Data are mean±s.e.m. (D) Lgr4KO spheroids derived from postnatal intestine (P15) grow normally and can be subcultured in BCM conditions. Scale bars: 100 µm.

The yield of fetal intestinal spheroids obtained by the EDTA Sato protocol decreases steadily during fetal life to reach zero shortly after birth (Fordham et al., 2013; Mustata et al., 2013). To explore the possibility that adult spheroids would be identical to fetal spheroids, but generated from cells more tightly attached to the matrix in postnatal tissue, we compared the culture medium requirement of both types of spheroids. Whereas, in our hands, fetal spheroids grow best in ENR-containing medium (EGF, noggin and Rspondin) (Sato et al., 2009), and depend completely on Rspondin and expression of the Lgr4 gene for survival (Mustata et al., 2013), adult spheroids survive and thrive in plain culture medium without the addition of growth factors (Fig. 2A-C). In long-term cultures, plain basal cell medium (BCM) allowed the maintenance of the clear spheroid phenotype, whereas in EN (EGF and noggin) or ENR they became dark and shrank (Fig. 2B,C) if not replated every 2 days. In addition, and contrary to fetal spheroids, Lgr4-deficient spheroids from P15 mice survive (Fig. 2D) and could be replated, which is consistent with their independence from exogenous Rspondin. Similar intestinal spheroids displaying a Wnt (Rspondin)-independent phenotype have been obtained from culture of isolated Bmi+ve ‘+ 4’ cells (Smith et al., 2018) or in models of intestinal regeneration *in vivo* (Nusse et al., 2018) or *ex vivo* (Yui et al., 2018). These studies constituted the first indication of a potential link between adult collagenase/dispase-derived spheroids and tissue regeneration.

### Adult spheroids are made of poorly differentiated intestinal epithelial cells with downregulation of CBC markers and expression of a regeneration program

**Fig. 3.**
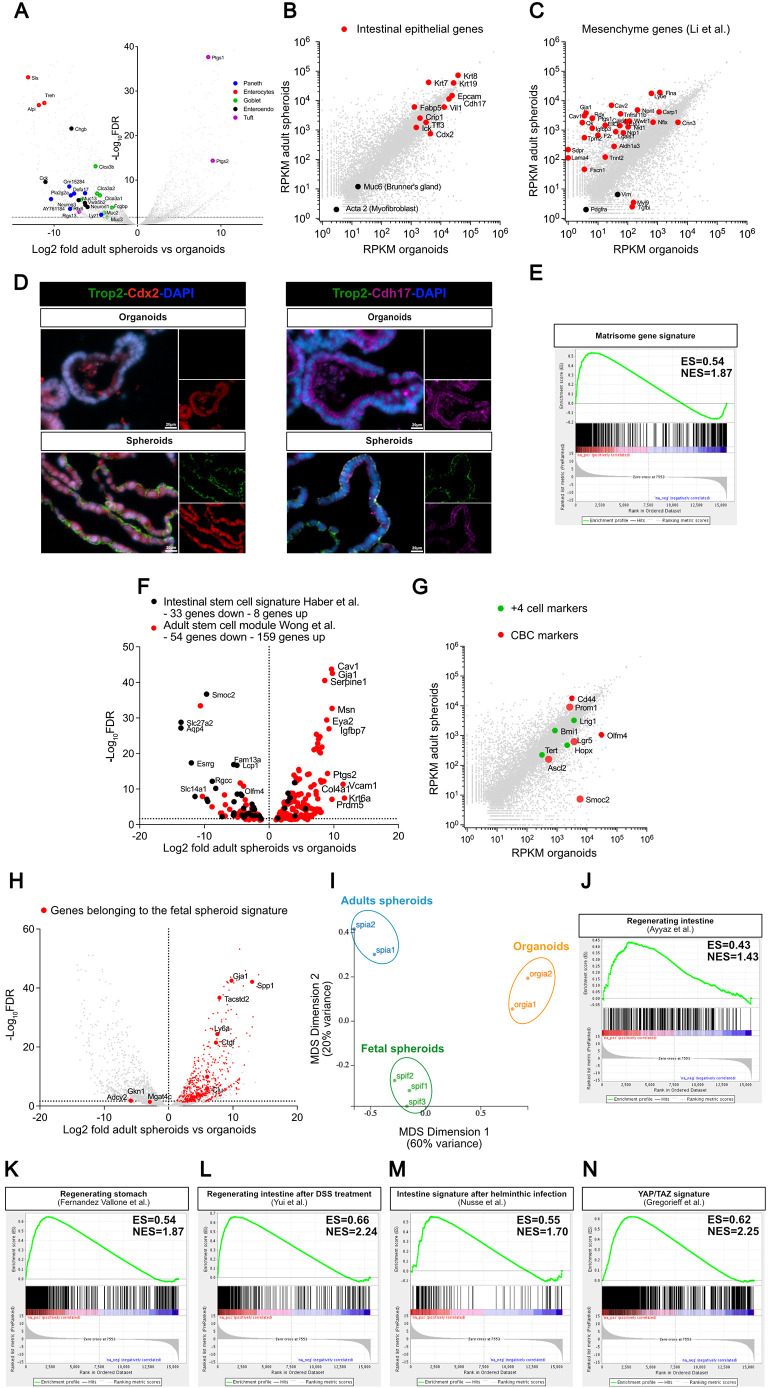
**Gene expression program of adult spheroids.** (A) Volcano plot showing that, compared to organoids, adult spheroid transcripts display partial downregulation of markers representative of all five intestinal differentiated cell types; to allow pertinent comparison, spheroids and organoids were cultured in the same EGF, noggin and Rspondin (ENR)-containing medium. (B) Logarithmic plot showing reads per kilobase per million (RPKM) measured for adult spheroids and organoids; red dots indicate expression of genes illustrating the intestinal epithelial nature of spheroids. (C) Logarithmic plot showing RPKM for adult spheroids and organoids; red dots indicate expression of genes belonging to the intestinal mesenchymal gene signature by Li et al. (2007). (D) Immunofluorescence showing Trop2-Cdx2 and Trop2-Cdh17 expression on spheroids and organoids, confirming the intestinal origin of adult spheroids. Scale bars: 20 µm. (E) Pre-ranked gene set enrichment analysis (GSEA) showing positive correlation between adult spheroid upregulated genes (versus organoids) and matrisome signature described by Naba et al. (2012). (F) Volcano plot showing upregulation of an ‘adult tissue stem cell signature’ (red dots indicate 54 genes downregulated and 159 genes upregulated) (Wong et al., 2008) and downregulation of an ‘intestinal stem cell signature’ (black indicates 33 genes downregulated and 8 genes upregulated) (Haber et al., 2017) in adult spheroids, compared to organoids (total number of genes modulated with an FDR<0.05 is 3329). (G) Logarithmic plot showing RPKM for adult spheroids and organoids; dots indicate ‘+4’ (green) or CBC (red) stem cell markers; with the exception of prominin and CD44, CBC markers were downregulated and ‘+4’ markers were expressed at levels similar to those in organoids. (H) Volcano plot of adult spheroid versus organoid transcriptomes, showing upregulation of the vast majority of genes belonging to the fetal spheroid signature (red dots) (Fernandez et al., 2016). (I) Multidimensional scaling plot (MDS) displaying relatedness between adult intestinal spheroids (blue), fetal intestinal spheroids (green) and intestinal organoids (orange). (J-N) Pre-ranked GSEAs showing the following: positive correlation between adult spheroid upregulated genes (versus organoids) and the gene signature of Clu+ve ‘revival stem cells’ from irradiated intestine (J; Ayyaz et al., 2019), regenerating stomach (K; Fernandez et al., 2016), regenerating intestine induced by dextran sulphate sodium (DSS) treatment in a murine model of colitis (L; Yui et al., 2018), the intestine following helminthic infection (M; Nusse et al., 2018); and Yap/Taz gene expression signature (N; Gregorieff et al., 2015). ES, enrichment score; NES, normalized enrichment score.

Bulk RNAseq demonstrated that adult spheroids display partial downregulation of genes specific for most intestinal lineages (Haber et al., 2017) [enterocytes (Sis, Alpi, Treh…), Goblet cells (Clca3a1, Clca3a2, Clca3b, Muc2, Muc3, Fcgbp…), Paneth cells (Defa-rs1, Defa17, Lyz1, Gm15284, AY761184…), enteroendocrine cells (Chgb, Neurod1, Neurog3, Vwa5b2…)] (Fig. 3A, Fig. S2A-E, Table S1). However, they are clearly intestinal in origin with lower but definite expression of Cdx2 (Fig. 3B,D), and robust expression of several transcripts enriched in (Vil1, Crip1, Ick, Epcam, Krt8, Krt19, Krt7) or highly specific for (Cdh17, Tff3) the intestinal epithelium (Fig. 3B,D). Together with their capacity to thrive and be replated in basal culture medium without added growth factors (Fig. 2B,C) – a characteristic not shared by pancreatic duct organoids (Huch et al., 2013) – high expression of Cdh17 and absence of expression of Muc6 (Fig. 3B) make it highly improbable that they could originate from contaminating pancreatic ducts or Brunners gland material.

Unexpectedly, given their epithelial phenotype, with the notable exceptions of vimentin and Pdgfra genes, these spheroids display strong upregulation of a set of genes expressed in intestinal mesenchyme (Li et al., 2007) (Fig. 3C), together with a matrisome gene signature (type 4 collagens, laminins, metalloproteases, fibronectin, Ctgf, cytokines, Pdgf…) (Naba et al., 2012) (Fig. 3E). All these characteristics were observed for spheroids cultured in ENR (that needed being replated every other day, see Fig. 2) and more so in spheroids stably cultured in basal medium (Table S1).

In agreement with their aptitude to be serially replated over long periods (26 replatings with maintenance of a stable phenotype) and to resist freeze/thaw cycles, adult spheroids express a set of upregulated genes overlapping significantly with an ‘adult tissue stem cell module’ [159/721 genes; q value 2.11 e^−94^) (Fig. S2F)]. However, they paradoxically display downregulation of several genes defining the ‘intestinal stem cell signature’, including the paradigmatic CBC markers (Lgr5, Ascl2, Smoc2 and Olfm4) (Fig. 3F,G; Fig. S2G) (Haber et al., 2017). Markers of ‘+4’ stem cells (Bmi1, Tert, Lrig1 and Hopx) were all expressed in adult spheroids, with no significant up- or downregulation compared to organoids (Fig. 3G) (Richmond et al., 2016).

Bulk RNASeq data confirmed the close relationship of adult spheroids with fetal spheroids (Fig. 3H, Fig. S2H-J). Out of the 692 genes constituting a fetal intestinal and gastric spheroid signature (Fernandez et al., 2016), only three were slightly downregulated, and 533 were upregulated in adult spheroids, including Ly6a (also known as Sca1), Tacstd2 (also known as Trop2), Gja1, Ctgf, Clu and Spp1 (Fig. 3H). However, despite their similarity, adult and fetal spheroids are clearly distinct (Fig. 3I), with adult spheroids showing 1169 and 768 genes up- or downregulated, respectively, when compared to fetal intestinal spheroids (Table S2). GSEA analyses for hallmarks comparing the adult and fetal spheroid transcriptomes, revealed highly significant upregulation of genes implicated in epithelial-mesenchymal transition (61 genes; FDR q value e^−51^), activation of the NFkB pathway (58 genes; FDR q value e^−48^) or inflammation (42 genes; FDR q value e^−28^), and stimulation by interferon gamma (39 genes; FDR q value e^−25^) (Table S3). Finally, GSEA analyses demonstrated striking similarities between the transcription program of adult spheroids and those observed in several models of regenerating gut (Ayyaz et al., 2019; Fernandez et al., 2016; Nusse et al., 2018; Yui et al., 2018) (Fig. 3J-M), with expression of a robust Yap/Taz gene signature (Gregorieff et al., 2015) (Fig. 3N).

The spheroid phenotype proved to be extremely stable. Attempts to break symmetry and to differentiate them *ex vivo* into organoids by culturing them in ENR containing a series of agents known to affect intestinal differentiation were unsuccessful (see legend to Fig. S3). In particular, Notch inhibition by DAPT, which allowed differentiation of fetal spheroids (Mustata et al., 2013), was ineffective (Fig. S3A). Despite preferential expression of several Wnt genes in spheroids and robust expression of Frizzled genes in both spheroids and organoids (Fig. S3B,C), inhibition of Wnt by the porcupine inhibitor Iwp2 was without effect on spheroids, while it strongly affected survival of organoids upon replating (Fig. S3D-G). Inhibition of the Yap/Taz pathway by verteporfin did not allow differentiation, but showed a dose-dependent negative effect on adult spheroid survival (Fig. S3H,I).

### Adult spheroids originate from activation of a regeneration program in cells following their release from the extracellular matrix by collagenase/dispase

Since adult spheroids were obtained in variable amounts and not from all mice, we initially hypothesized that they originated from regions of the intestine of some animals in which regeneration takes place following minimal ‘physiological’ injuries. However, using a new two-layer sandwich protocol described by the Lutolf group (Bues et al., 2020 preprint), we reproducibly obtained adult spheroids from all mice (Fig. S4A-C). In this protocol, cells dissociated from the mesenchyme by collagenase/dispase are first allowed to spread on the surface of matrigel, before being covered by a second matrigel layer. Considering the known relation between matrix interaction and stem cell quiescence and/or activation (Gilbert et al., 2010; Machado et al., 2021; Montarras et al., 2005), our interpretation is that spreading of cells at the origin of spheroids on the first layer of matrigel might mimic the effects of tissue injury more closely than abrupt culture in matrigel. The spheroids obtained with the dome and sandwich protocols were very similar regarding transcriptome and culture medium requirements (Fig. S4B,D,E).

In most regenerating conditions of the intestinal epithelium, after initial activation of a Yap/Taz genetic program, new Lgr5+ve CBCs are generated. We propose that spheroids are made from cells that become ‘frozen’ in a stable Yap/Taz regenerating-like phenotype, following their release from the matrix by collagenase/dispase treatment. Together, these results suggest the existence of atypical stem cells, tightly attached to the extracellular matrix, that activate a regeneration program when they are severed from their normal environment by collagenase/dispase treatment and cultured in matrigel.

### Organoids and adult spheroids originate from separate developmental intestinal lineages

**Fig. 4.**
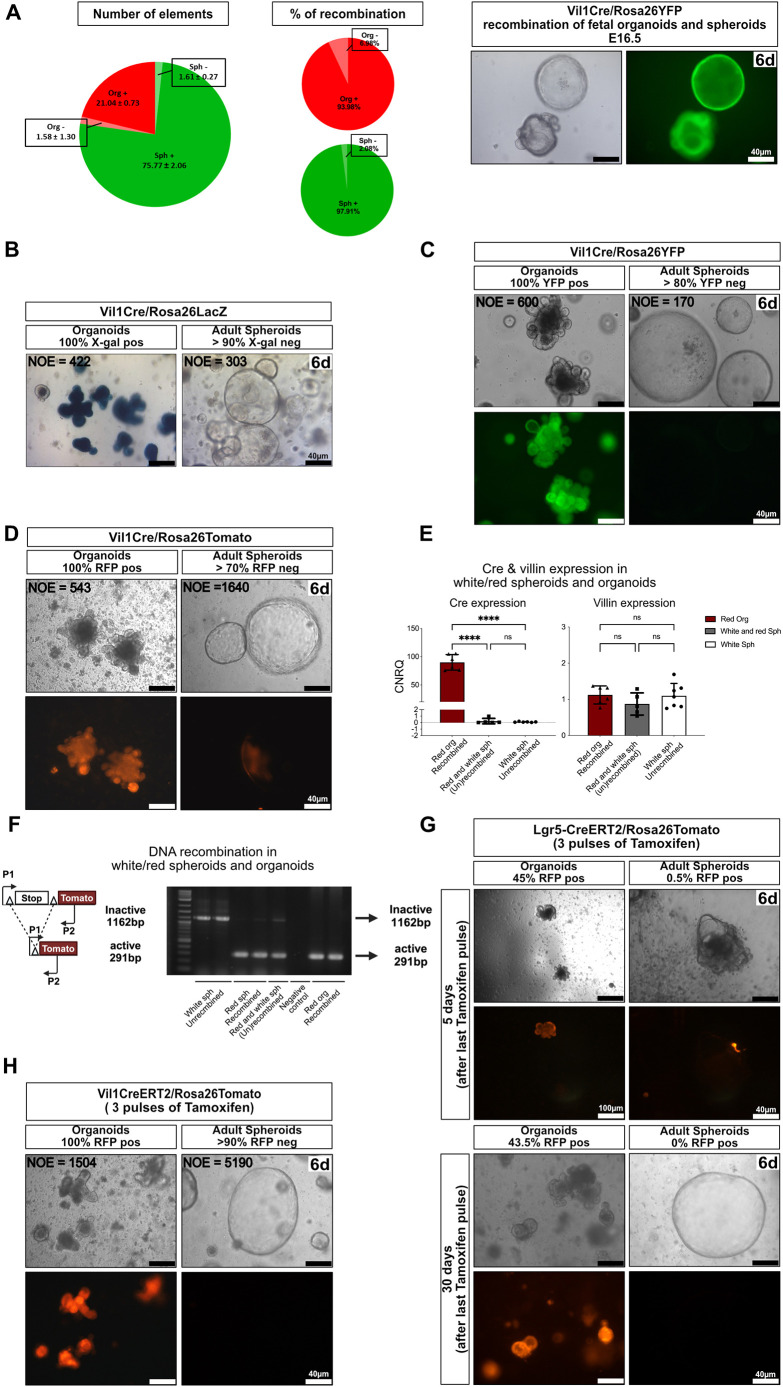
**Organoids and adult spheroids originate from separate developmental intestinal lineages.** (A) Proportion of elements and percentages of recombined fetal organoids and spheroids from E16.5 Vil1Cre/Rosa26YFP mice (left); representative images (right) at day 6 of initial seeding (*n*=3). (B-D) Representative images showing absence of recombination in spheroids from Vil1Cre mice using Rosa26LacZ (*n*=2) (B), Rosa26YFP (*n*=7) (C) or Rosa26Tomato (*n*=4) (D) reporters 6 days after initial seeding. (E) qRT-PCR of Cre (expressed as CRNQ) and villin (expressed relative to level in organoids) measured in organoids and in Tom+ve (red/recombined) and Tom-ve (white/unrecombined) spheroids. Data are mean±s.e.m. ns, not significant; ****P<0.0001 (one-way ANOVA test with Tukey‘s multiple comparison tests). (F) PCR strategy targeting the Rosa26Tomato locus showing the absence of recombination in white spheroids prepared from Vil1Cre/Rosa26Tomato mice. (G) Absence of recombination in spheroids derived from Lgr5CreERT/Rosa26Tomato mice after three pulses of tamoxifen (*n*=2 for short chase; *n*=3 for long chase). (H) Absence of recombination in spheroids cultured from Vil1CreERT2/Rosa26Tomato mice after three pulses of tamoxifen (*n*=4). Scale bars: 40 µm; 100 µm in upper left images in G. Labels in the upper right corner of the images refer to the days after initiation of cultures. NOE, number of elements counted for each condition.

Given expression of both mesenchymal and epithelial markers in adult spheroids (Fig. 3B-E), we wanted first to ensure that they belong to the epithelial intestine lineage. To achieve this, we used the Tg(Vil1Cre)997Gum/J mouse line (Madison et al., 2002), expressing a Cre recombinase transgene specifically in the intestinal epithelium, starting at E12.5. To our surprise, whereas close to 100% of EDTA-organoids and spheroids generated from E16.5 Vil1Cre/Rosa26 mice were positive for reporter expression, as expected (Fig. 4A), the vast majority of the adult spheroids were negative (70-90%) (Fig. 4B-D), despite displaying robust expression of villin (Figs 3B, 4E). This difference is in strong contrast to the low level of mosaicism (at most a few percent) displayed by this mouse line (Madison et al., 2002), which we interpret as an indication that the absence of Vil1Cre expression constitutes a potent tracing opportunity. To explore the reasons for this observation, we quantified Cre transcripts in spheroids and organoids from Vil1Cre/Rosa26Tomato mice. This demonstrated robust Cre expression in organoids and very low or absence of Cre mRNA in both ‘white’ and ‘red’ spheroids (Fig. 4E), the latter probably related to the exquisite sensitivity of the Rosa26 Ai9 Tomato reporter to traces of recombinase (Liu et al., 2013). Further confirming these results, direct measurement of recombination at the Rosa26Tomato locus showed absence of recombination in white spheroids, when organoids were 100% recombined (Fig. 4F). In addition, no significant tracing of spheroids was obtained from Lgr5CreERT2/Rosa26Tomato animals 5 days or 30 days after three pulses of tamoxifen (Fig. 4G). This indicates that, while expressing low but detectable levels of Lgr5 upon culture (Fig. 3G), adult spheroids originate from a Lgr5-negative developmental linage.

Since adult spheroids display robust expression of villin (Fig. 3B), one must conclude that, contrary to the endogenous villin promoter, the 12.4 kb transgene fragment in the construct stays inactive in the whole cell lineage at the origin of adult spheroids. Similarly, tamoxifen administration to Vil1CreERT2/Rosa26YFP or Vil1CreERT2/Rosa26Tomato mice, which harbour a shorter villin promoter fragment (9 kb) (el Marjou et al., 2004), also yielded 100% recombined organoids and close to 100% un-recombined spheroids (Fig. 4H). These experiments suggest that transcription factors must be acting differently at the endogenous villin gene promoter/enhancers in organoids and adult spheroids, respectively. Results from AtacSeq experiments give support to this hypothesis, showing different patterns of open chromatin in the vicinity of the endogenous villin gene in spheroids and organoids (Fig. S5). We interpret this as meaning that different cis-regulatory sequences are used for endogenous villin gene expression in organoids and spheroids. We conclude from the differential behaviour of the Vil1Cre transgenes in adult spheroids and organoids that the two structures originate from different developmental lineages.

### Adult spheroid-generating cells are involved in regeneration of the intestinal epithelium following injury

Since non-recombined cells are exceedingly rare in the intestinal epithelium of Vil1Cre/Rosa26Tomato mice (Madison et al., 2002) under homeostatic conditions, our results suggest that collagenase/ dispase treatment of intestinal tissue followed by 3D culture in matrigel triggers proliferation of rare quiescent cells belonging to a hitherto undescribed intestinal lineage. Collagenase/dispase solution would act as a proxy for injury, and adult spheroids would correspond to a stable avatar of intestinal regenerating cells, trapped in a state compatible with unlimited *ex vivo* culture. If this hypothesis holds true, we reasoned that we could use the absence of recombination in Vil1Cre/Rosa26Tomato intestine as a tool to trace the possible involvement of adult spheroid-generating cells in intestinal regeneration. We tested this hypothesis in two injury models.

**Fig. 5.**
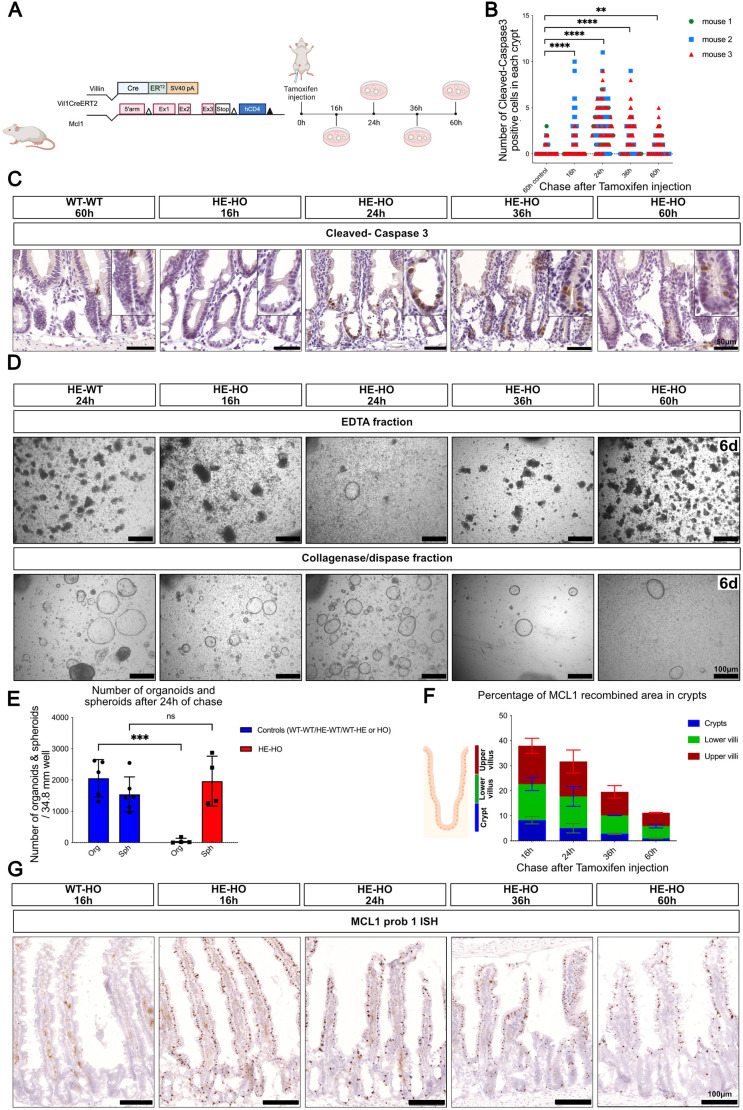
**Regeneration of Mcl1-ablated intestine originates from cells escaping recombination triggered by VilCreERT2.** (A) Schematic summary of the experiment. (B) Number of caspase 3-positive cells in crypt cells at various time after tamoxifen injection (*n*=3). **P=0.0028, ****P<0.0001 (two-way ANOVA test with Dunnett‘s multiple comparisons test). (C) Representative pictures of crypts with caspase 3-positive cells (WT-WT, wild-type mice; HE-HO, Vil1CreERT2 heterozygotes, homozygote for Mcl1^fl/fl^). (D) Representative images showing the yield of organoids (EDTA fraction panels) or spheroids (collagenase-dispase fraction panels) at various time points after tamoxifen administration. Cultures are shown 6 days after plating. (E) Quantification of spheroids and organoids per well, 24 h after tamoxifen injection; each symbol illustrates results from one mouse (*n*>4). Data are mean±s.e.m. ns indicates *P*>0.05, ***P<0.001 (two-way ANOVA test with Šídák's multiple comparisons). (F) Percentage of epithelial surface containing Mcl1^fl/fl^ recombined cells, along the crypt-villus axis at various times after tamoxifen administration, *n*=2-7 mice (see also Fig. S5). Data are mean±s.e.m. (G) Representative images of intestinal epithelium showing Mcl1 RNAscope signals at various times after tamoxifen administration. Paradoxically, recombined cells show strong Mcl1-RNAscope signals (see text and Fig. S6). Scale bars: 40 μm in C; 100 μm in D and G. Schematics created in BioRender by Hadefi, A., 2025. https://BioRender.com/a95a941. Republished with permission.

In the first model, the ubiquitously expressed anti-apoptotic gene *Mcl1* was ablated in the intestinal epithelium of Vil1CreERT2/Mcl1^fl/fl^ mice (Vikstrom et al., 2010) (Fig. 5A). A wave of apoptosis was observed in the crypts of animals treated with tamoxifen, with a maximum peaking at 24 h (Fig. 5B,C). When the tissue was processed for organoid and spheroid production, no organoids could be obtained from tamoxifen-treated animals at 24 h, indicating effective ablation of CBCs. Unexpectedly, rare spheroids were cultured from the EDTA fraction 24 h after tamoxifen treatment. Spheroids were obtained from collagenase/ dispase fractions irrespective of tamoxifen treatment (Fig. 5D,E), which fits with the hypothesis that they originate from cells belonging to a lineage that is different from CBCs. Looking for regenerating crypts possibly growing from this non-recombined lineage, we performed RNAscope *in situ* hybridization (ISH) with two Mcl1 probes, which yielded unexpected results. Removal of the floxed segment of the Mcl1 gene resulted in a strong increase of the ISH signals obtained in the intestinal epithelium with both probes (Fig. S6). This must result from the removal of a transcriptional silencer from the gene or from stabilization of the mRNA transcribed from the recombined gene (or from both). Whatever the explanation, this increase in ISH signal constitutes a sensitive and convenient proxy for recombination, allowing the presence of un-recombined regenerating crypts to be examined. When performed 16 h, 24 h, 36 h and 60 h after tamoxifen administration to Vil1CreERT2/Mcl1^fl/fl^ mice, *Mcl1* ISH showed a wave of un-recombined cells progressing from the crypts towards the villi (Fig. 5F,G). Given the absence of organoid-generating cells in tamoxifen-treated Vil1CreERT2/Mcl1^fl/fl^ mice at 24 h (Fig. 5D,E), it seems that recombination efficiently ablates most CBCs and that, indeed, regeneration starts from un-recombined cells. However, in this model, we cannot differentiate between the hypothesis that the wave of un-recombined crypts would originate from CBC or TA cell escapers, rather than from a specific lineage. We therefore turned to a second model of injury involving constitutive Vil1Cre expression.

**Fig. 6.**
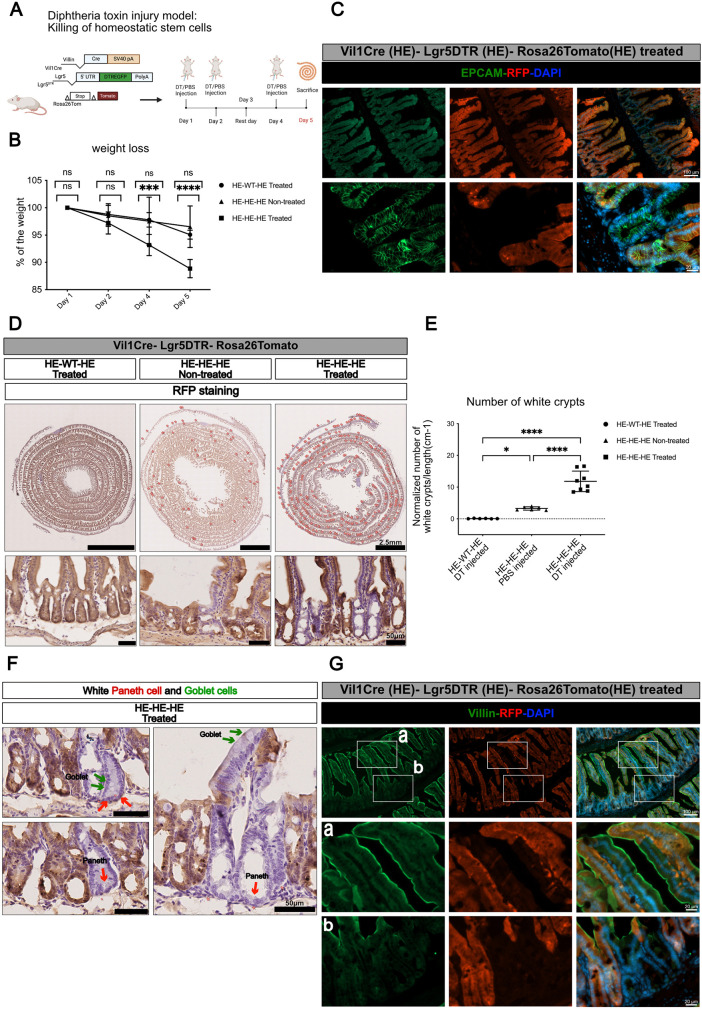
**Contribution of Vil1Cre-negative cells to epithelial regeneration after CBC ablation.** (A) Schematic summary of the experiment. (B) Kinetics of weight loss of animals after diphtheria toxin (DT) treatment (*n*=6-8 mice in each group). ns indicates *P*>0.05, ***P<0.001, ****P<0.0001 (two-way ANOVA test with Dunnett‘s multiple comparisons test). (C) Immunofluorescence showing a RFP-negative (un-recombined) patch of epithelium in the small intestine of a Vil1Cre/Lgr5DTR/Rosa26Tomato mouse treated with DT. (D,E) Quantification of RFP-negative crypts in Swiss rolls from animals treated or not with diphtheria toxin. HE-WT-HE, VilCre/Rosa26Tomato double heterozygotes; HE-HE-HE, Vil1Cre/Lgr5DTR/Rosa26Tomato triple heterozygotes. The crypts appearing as ‘white’ after RFP immunohistochemistry (bottom row) are marked on the rolls (top row), counted and the results normalized to the length of the roll. Each symbol illustrates results from one animal (E); all samples were from mice euthanised at day 5 (see A). *P=0.0430, ****P<0.0001 (one-way ANOVA test with Tukey‘s multiple comparison tests). (F) Higher magnification showing presence of ‘white’ Paneth (red arrows) and goblet (green arrows) cells in un-recombined epithelium patches. (G) Immunofluorescence showing villin expression in villi (a) or crypts (b) of a RFP-negative (un-recombined) patch of epithelium in the small intestine of a Vil1Cre/Lgr5DTR/Rosa26Tomato mouse treated with DT. Scale bars: 50 µm in C, D (bottom row), F, G); 2.5 mm in D (top row). Schematics created in BioRender by Hadefi, A., 2025. https://BioRender.com/a95a941. Republished with permission.

In the second model, CBCs are ablated by diphtheria toxin (DT) administration to mice expressing the human diphtheria toxin receptor gene under control of Lgr5 regulatory regions (Lgr5-DTR knock-in mice; Tian et al., 2011) (Fig. 6A). After three pulses of toxin administration over a period of 5 days, Vil1Cre/Lgr5-DTR/Rosa26Tomato triple heterozygotes mice lost weight (Fig. 6B) and multiple Tomato-negative clones were observed all along the small intestine, some of which extended to the tip of villi and encompassed morphologically identified Paneth and goblet cells (Fig. 6C-F). Despite being Tomato negative (i.e. Vil11Cre-negative), and similar to adult spheroids, these regenerating clones expressed villin strongly in villi (Fig. 6Ga). At the crypt level, villin expression was low, irrespective of Tomato expression (Fig. 6Gb). Tomato-negative crypts were virtually absent in Vil1Cre/Rosa26Tom double heterozygote mice treated with the toxin (Fig. 6D,E). Unexpectedly, untreated Vil1Cre/Lgr5-DTR/Rosa26Tomato triple heterozygotes displayed more un-recombined crypts than did the treated Vil1Cre/Rosa26Tomato double heterozygotes, although on average three times less than treated ones (Fig. 6D,E). This suggests that either monoallelic expression of Lgr5 or ectopic expression of the diphtheria toxin receptor in CBCs causes a low-grade chronic injury. The absence of increase of Tomato-negative crypts in two lines of mice with monoallelic expression of Lgr5 in CBCs (Fig. S7A-D) points to a direct effect of ectopic DTR expression. Although neglected by the many groups using DTR-expressing mice, an effect of HB-EGF (the DTR) has been documented in the intestine, where it causes inflammation and stimulates cell proliferation (Chen et al., 2010). In line with this hypothesis, bulk RNAseq demonstrates activation in untreated Lgr5-DTR mice of a program overlapping that of adult spheroids (Fig. S7), with a proportion of genes commonly up- or downregulated, belonging to Wong adult tissue stem cell module (Wong et al., 2008; Fig. 3F) or to differentiated intestinal epithelium (Fig. S7E-I), respectively.

**Fig. 7.**
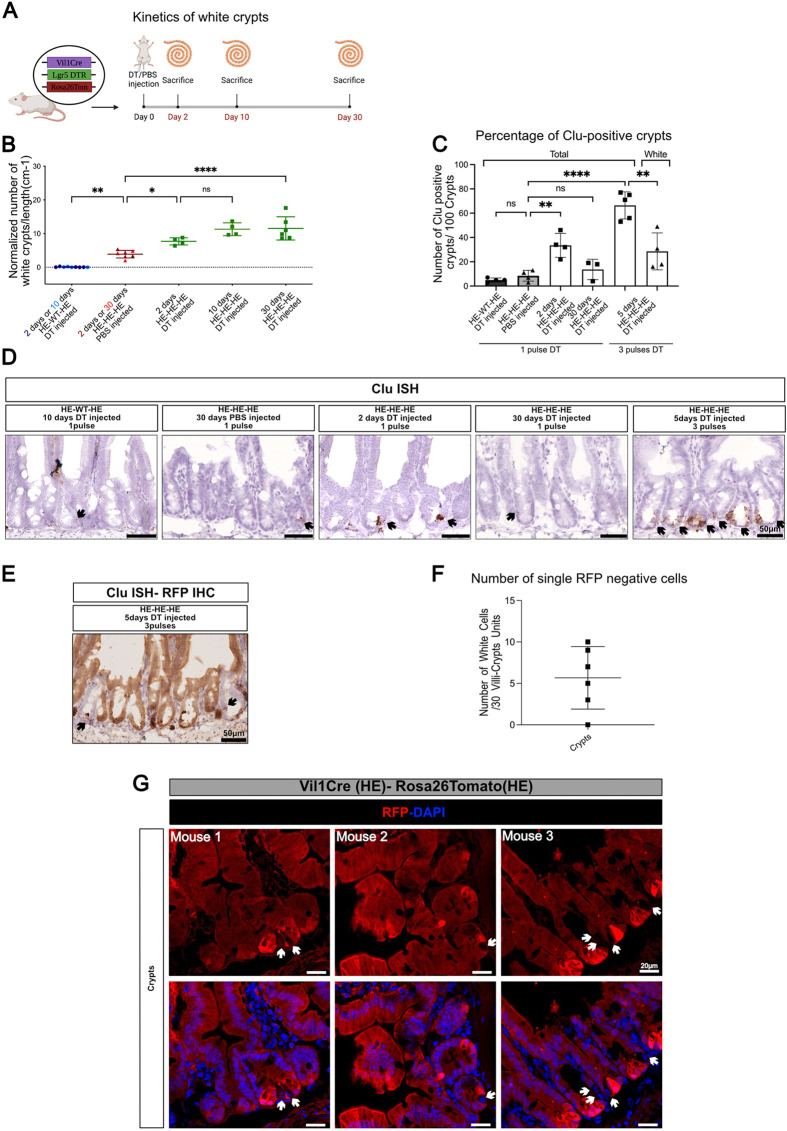
**Kinetics of un-recombined crypt generation following CBC ablation.** (A) Schematic summary of the experiment. (B) Kinetics of un-recombined (white) crypt generation after a single injection of diphtheria toxin (DT). HE-HE-HE, Vil1Cre/Lgr5DTR/Rosa26Tomato triple heterozygotes; HE-WT-HE, Vil1Cre/Rosa26Tomato double heterozygotes. Each symbol illustrates results from one animal. Data are mean±s.e.m. ns indicates *P*>0.05, *P<0.02, **P<0.01, ****P<0.0001 (one-way ANOVA test with Tukey‘s multiple comparison tests). (C) Percentage of total or un-recombined (white) Clu-positive crypts by RNAscope *in situ* hybridization at different time points after one pulse (see A and B) or three pulses of tamoxifen (see Fig. 6A,E). Data are mean±s.e.m. ns indicates *P*>0.05, **P<0.01, ****P<0.0001 (one-way ANOVA test with Tukey‘s multiple comparison tests). (D) Representative images of intestinal epithelium showing the presence of Clu-positive cells in crypts after tamoxifen administration at different time points. (E) Representative image of intestinal epithelium showing the presence of Clu-positive cells (arrows) in red and white crypts. (F) Number of single Tomato-neg cells in 30 crypts in Vil1Cre/Rosa26Tomato double heterozygote mice; each symbol illustrates results from one mouse (*n*=6). Data are mean±s.e.m. (G) Representative images of immunofluorescence showing Tomato-neg single cells in crypts (arrows). Schematics created in BioRender by Hadefi, A, 2025. https://BioRender.com/q28z760. Republished with permission.

The kinetics of appearance of newly formed un-recombined (‘white’) crypts was studied after a single pulse of DT (Fig. 7A). This demonstrated an increase at 48 h, with further a increase at day 10 and stable maintenance at day 30. The presence of newly formed white crypts 1 month after toxin administration indicates that the Vil1Cre-negative lineage is developmentally stable and does not turn on the transgene during differentiation of the various epithelial lineages occurring after regeneration (Fig. 7B). The relationship between white crypt generation and appearance of Clu-positive revival cells (Ayyaz et al., 2019) was then explored. In agreement with others and similar to what happens in the irradiation model (Ayyaz et al., 2019; Yuan et al., 2023), Clu-positive cells were rare in crypts of untreated mice and their number transiently increased 48 h after a single pulse of DT, and more so after three pulses of DT (Fig. 7C,D,E). Clu-positive cells were less frequently observed in white crypts (see ‘Total’ versus ‘White’ in Fig. 7C). This fits with the hypothesis that Clu expression marks acutely regenerating crypts and that a proportion of the white crypts not showing Clu expression corresponds to those that underwent regeneration at an undetermined time in the past, due to DTR expression in CBCs. This observation indirectly suggests that LGR5-DTR is not expressed in these ‘chronically white’ crypts.

Altogether, our results strongly suggest that the same Vil1Cre-negative cell lineage is at the origin of adult spheroids and a fraction of regenerating crypts observed in the diphtheria toxin model of injury. The actual proportion of crypts regenerating from this lineage is difficult to evaluate. Indeed, given the extreme sensitivity of the Rosa26Tomato reporter to traces of recombinase (Liu et al., 2013; Fig. 4E), it is likely that using absence of recombination as a criterion leads to an underestimation of their number.

### Cells at the origin of the Vil1Cre-negative lineage are quiescent Olfm4-positive cells

**Fig. 8.**
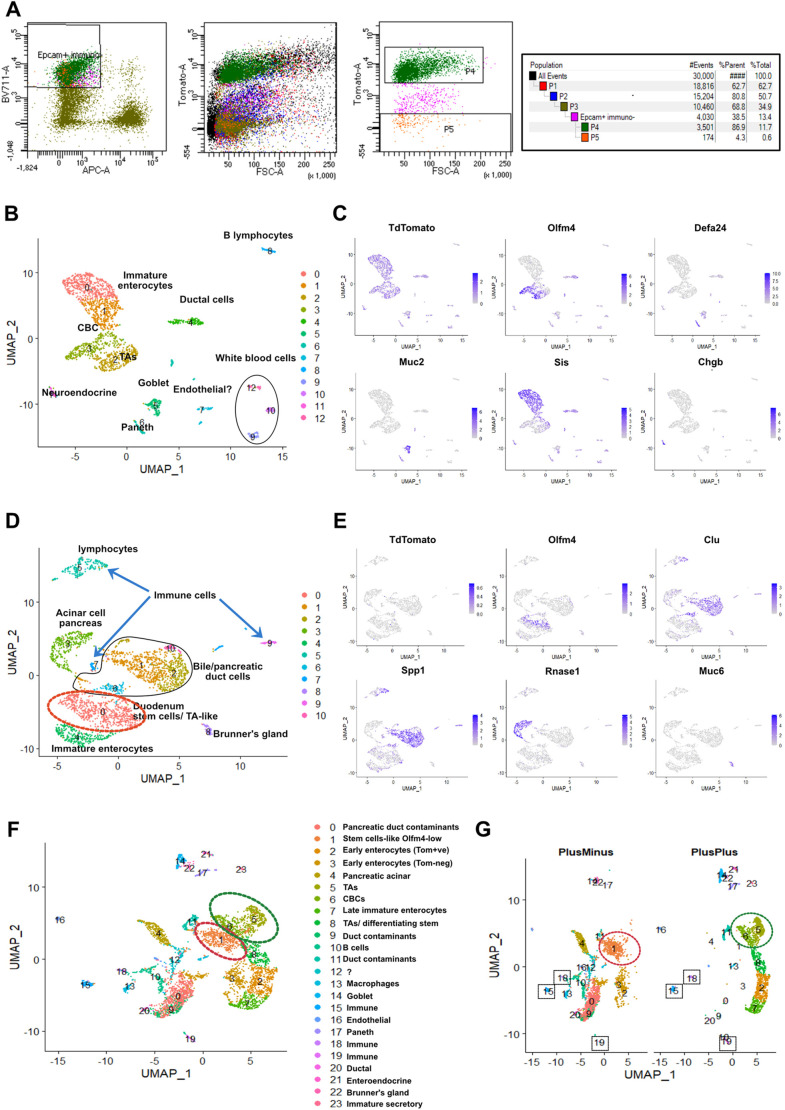
**scRNAseq of Epcam-positive-Rosa26Tomato-positive (PlusPlus) and Epcam-positive-Rosa26Tomato-negative cells (PlusMinus).** (A) Fluorescent sorting of PlusPlus cells (P4 window) and PlusMinus cells (P5 window). (B) UMAP representation of scRNAseq of 2154 PlusPlus cells showing 12 clusters identified by their differentially expressed genes (DEGs). (C) The same UMAP showing expression of Tdtomato, Olfm4, Defa24, Muc2, Sis and Chgb. (D) UMAP representation of scRNAseq of 2650 PlusMinus cells showing 10 clusters identified by their DEGs. (E) The same UMAP showing expression of Tdtomato, Olfm4, Clu, Spp1, Rnase1 and Muc6. (F) UMAP representations of similar number of cells from PlusPlus and PlusMinus samples after merging data analysis in Seurat, with indication of the cell types identified from their DEG lists. Olfm4-positive clusters are indicated by a dotted outline. (G) The same data displayed according to their PlusPlus or PlusMinus origin, with indication of overlapping contaminants (squares). (H) The same UMAP showing expression of Olfm4 and Mki67. (I) Violin plots showing expression level of canonical transcript of CBCs (Olfm4, Smoc2 and Slc12a2), +4 (Hopx) and the Wnt target genes *Axin2* and *Mki67* in all the clusters involved in merged data analysis.

Un-recombined cells are exceedingly rare in crypts of Vil1Cre/Rosa26Tomato double heterozygotes (Fig. 7F,G). In an attempt to characterize them, Epcam+ve/Rosa26Tomato-negative cells (‘PlusMinus’ cells, hereafter) were purified by FACS from the tissue fraction giving rise to adult spheroids (Fig. 8A). This fraction is made of what remains after EDTA extraction of crypts from the total intestine (see Fig. 1B). Microscopic inspection reveals that it is essentially made of mesenchyme delineating ghosts of crypts (Fig. S1A). Effectively, PlusMinus cells represented a small proportion of the cells in this fraction (0.6%, Fig. 8A), which were then subjected to scRNAseq, together with Epcam-positive and Rosa26Tomato-positive cells from the same fraction (‘PlusPlus’ cells, hereafter).

**Fig. 9.**
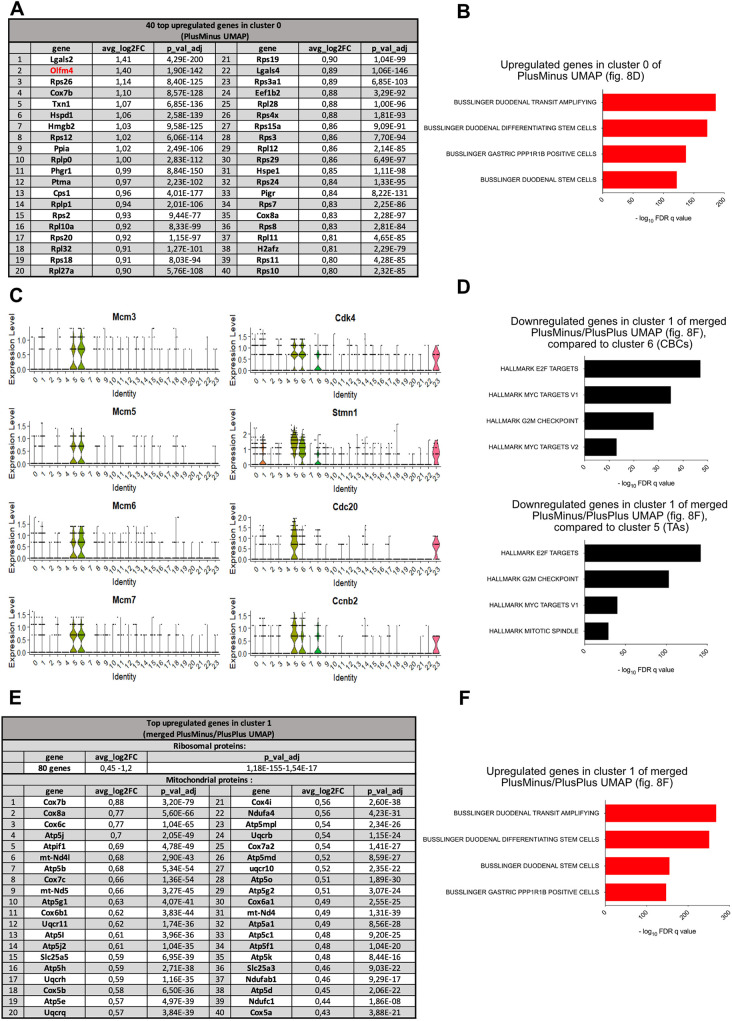
**Cells at the origin of the VilCre-negative lineage are quiescent Olfm4-positive cells.** (A) Table showing 40 upregulated genes in cluster 0 of PlusMinus (Fig. 8D) sorted by log2 fold change. (B) GSEA MolSig results for biological cell types (C8) associated with the upregulated genes in cluster 0. (C) Expression levels of various genes implicated in cell cycle and mitosis across the merged datasets (Fig. 8F). (D) GSEA MolSig (hallmarks) results related to downregulated genes in cluster 1 of the combined PlusPlus and PlusMinus merged datasets (Fig. 8F), in comparison to cluster 6 (CBCs) and cluster 5 (TAs). (E) Table showing 40 upregulated genes in cluster 1 of merged datasets (Fig. 8F). (F) GSEA MolSig results for biological cell types (C8) associated with the upregulated genes in cluster 1 of the merged PlusPlus and PlusMinus datasets (Fig. 8F).

PlusPlus cells likely originate from crypts that were not released from their mesenchymal niche by EDTA. The Uniform Manifold Approximation and Projection (UMAP) plot of 2154 PlusPlus cells displayed the expected TdTomato-positive clusters of CBCs (cluster 2, in Fig. 8B,C), TAs, Paneth cells, precursors of enterocytes, Goblet and enteroendocrine cells, in addition to minor contaminants (Fig. 8B,C). The UMAP plot of 2650 PlusMinus cells was more complicated to interpret due to the presence of more contaminants (Fig. 8D): (1) Epcam-positive TdTomato-negative cells originating from Brunners glands (Muc6-positive cluster 8); (2) pancreatic tissue sticking to the duodenum (Clu-positive, Spp1-positive ductal cells, clusters 1, 2, 6 and 10; Fig. 8D,E); (3) Epcam-negative cells having escaped FACS selection, coming from pancreatic acini (Rnase1-positive cluster 3); and (4) immune cells (clusters 5, 7 and 9) (Fig. 8D,E). Nevertheless, the major cluster of PlusMinus cells (cluster 0) presents characteristics of epithelial duodenal stem cells, being Olfm4 positive and displaying abundant ribosomal protein transcripts (Fig. 8D,E and Fig. 9A,B). They represent 71% (659 cells) of the 930 bona fide intestinal epithelial cells [cluster 0 (659 cells)+cluster 4 (271 cells)]. In order to avoid normalization biases and compare transcriptomes of PlusPlus and PlusMinus cells more accurately, we merged data from the two samples and analysed the resulting UMAP (Fig. 8F,G). The presence of common contaminants (e.g. clusters 15, 18 and 19, Fig. 8G) demonstrates absence of significant batch effects in the merged analysis. If we concentrate on Olfm4-positive clusters (stem cell-like, see dotted ellipses in Fig. 8F,G), we observe that those originating from PlusMinus (cluster 1 in the merged plot) and PlusPlus [clusters 6 (CBCs) and 5 (TAs)] samples do not overlap (Fig. 8F,G). Several canonical transcripts of CBCs, including Olfm4 (Smoc2, Slc12a2 and Cdca7), are present but strongly downregulated in cluster 1, as are the Wnt target genes *Axin2* and *Mki67* (Fig. 8H,I). Of note, Hopx (Fig. 8I) and the others markers of the elusive ‘+4’ cells (Tert, Bmi1, Lrig1, not shown) are not detected in cluster 1. This suggested that cells in cluster 1 are related, but different from CBCs, and might be quiescent. Direct comparison of genes differentially expressed between cluster 1 and clusters 5 and 6 confirmed this hypothesis, with downregulation of genes controlling cell cycle and mitosis (Mcm3,5,6,7, Cdk4, Ccnd2, Stmn1…) (Fig. 9C,D).

From these results, we conclude that the Vil1Cre-negative lineage at the origin of adult spheroids and cells contributing to epithelial regeneration is made of quiescent stem cells sharing some characteristics with CBCs. The search for specific markers that would allow identification of these cells in the homeostatic intestinal epithelium yielded no results. Inspection of the differentially upregulated genes in these cells [cluster 0 of the PlusMinus UMAP (Fig. 9A) or cluster 1 of the merged UMAP (Fig. 9E)] revealed mainly the list of ribosomal protein genes characteristic of CBCs and, unexpectedly for quiescent cells, genes encoding cytochrome oxidase subunits, mitochondrial ATP synthase subunits and ATPase inhibiting factor (Fig. 9A,E).

## DISCUSSION

In vertebrates, steady-state maintenance and regeneration of tissues after injury is believed to follow two possible models: (1) a hierarchical model involving rarely dividing stem cells that generate progenitors with specific differentiation potentials (e.g. in haematopoiesis) (Seita and Weissman, 2010); and (2) a plasticity model characterized by the ability of committed or differentiated cells to de-differentiate, proliferate and reconstitute the various cell types (Wells and Watt, 2018). In the mammalian intestine, after the initial suggestion of a hierarchical model involving +4 rarely dividing label-retaining cells (Marshman et al., 2002), the identification of crypt base columnar cells (Cheng and Leblond, 1974) as actively dividing Lgr5+ve stem cells (Barker et al., 2007) changed this view. While Lgr5+ve cells were shown to be dispensable for daily epithelium maintenance (Tian et al., 2011), they are obligate actors of epithelial regeneration following, for example, irradiation (Metcalfe et al., 2014). According to current views, when the intestine is exposed to harmful stress, +4 quiescent cells, but also transit-amplifying progenitors or differentiated cells, acquire, or re-acquire, Lgr5+ve stem cell characteristics and reconstitute the epithelium. In addition, reciprocal interconversion of +4 and Lgr5+ve cells has been demonstrated, leading to a model characterized by extensive two-way differentiation plasticity (Clevers and Watt, 2018; Yousefi et al., 2017).

There is no question that all the above phenomena can take place following epithelial damage, as they were convincingly demonstrated by lineage-tracing experiments. However, their co-existence in, or relative contribution to, a given regenerating situation have not been assessed (Bankaitis et al., 2018; Buczacki, 2019). A role for Bmi1+ve cells in regeneration post-irradiation has been firmly established (Yan et al., 2012), but their heterogeneity and relation to Hopx+ve and Lgr5+ve cells (Beumer and Clevers, 2016; Li et al., 2016; Yousefi et al., 2017) made it difficult to identify precisely the cell(s) at the origin of the regeneration process.

Our results identifying a cell lineage at the origin of stable adult spheroids produced after collagenase/dispase dissociation of intestinal tissue casts new light on intestinal regeneration. These cells are not traced by a Vil1Cre transgene that is expressed from E12 in the main Lgr5-positve epithelial lineage, nor by Lgr5CreERT. Spheroids similar to those described in our study have been obtained from the intestine of mice infested with helminths (Nusse et al., 2018) or in an *ex vivo* model of tissue regeneration (Yui et al., 2018). This strongly suggests that they represent avatars of regenerating cells, ‘frozen’ in a stable culture-compatible state. This conclusion is strengthened by the expression of a common fetal-like genetic program (Mustata et al., 2013) and a strong Yap/Taz signature, both characteristics reportedly associated with intestinal regeneration (Moya and Halder, 2019; Yui et al., 2018), and by the similarity of their transcriptome with that of ‘revival stem cells’ (Ayyaz et al., 2019). Following ablation of CBCs in the diphtheria toxin injury model, Vil1Cre-negative crypt-villus units transiently expressing the revival cell marker Clu (Ayyaz et al., 2019) are generated, demonstrating the implication of this previously unreported intestinal lineage in intestinal regeneration.

Constitutive ablation of the anti-apoptotic gene Mcl1 in the intestinal tract of Vil1Cre/Mcl1^fl/fl^ mice has been shown to cause chronic apoptosis and inflammation in crypts, with associated hyperproliferation and dedifferentiation leading to the development of malignant tumours (Healy et al., 2020). A similar, but acute, inflammatory phenotype was observed in tamoxifen-treated Vil1CreERT2/Mcl1^fl/fl^ mice. Interestingly, whereas the phenotype was homogenous in the induced mouse model (Vil1CreERT2), it was strongly mosaic in the constitutive version (Vil1Cre), with areas of normal histology that retain expression of the *Mcl1* gene (Healy et al., 2020). Since both the Vil1Cre and Vil1CreERT2 transgenes are reportedly expressed throughout the intestinal epithelium (el Marjou et al., 2004; Madison et al., 2002), this observation is compatible with our hypothesis that a Vil1Cre- (or Vil1CreERT2)-negative intestinal lineage could be at the origin of regenerating intestinal cells. Our observation of a wave of un-recombined cells following tamoxifen injection to Vil1CreERT2/Mcl1^fl/fl^ mice could represent regeneration from such a pre-existing lineage. However, despite efficient recombination taking place in the Vil1CreERT2/Mcl1^fl/fl^ model (Healy et al., 2020; Fig. 5), we cannot exclude the possibility that it corresponds to proliferation of cells that have simply escaped recombination.

Despite an active controversy about the respective roles of quiescent, committed, differentiated or Lgr5-positive cells in intestinal regeneration (Bankaitis et al., 2018; Buczacki, 2019), there seems to be a consensus about their common belonging to the Lgr5-positive developmental lineage (Ayyaz et al., 2019; Nusse et al., 2018; Yui et al., 2018). Our observation that adult spheroids are not traced by Vil1Cre, nor by Lgr5CreERT, and that regenerating clones after CBC ablation are similarly not traced by the Vil1Cre transgene, demonstrates that at least a fraction of regenerating crypts originate from a Lgr5-independent lineage.

Identification of cells belonging to this lineage in the homeostatic intestinal epithelium pointed to cells sharing some characteristics with CBCs (Olfm4-positive, abundance of ribosomal protein transcripts) but with downregulation of proliferative markers, suggestive of a quiescent state. Participation of quiescent stem cells to intestinal regeneration has been documented for years (Scoville et al., 2008). They were coined ‘label-retaining cells’ from their aptitude to stably incorporate DNA tracers or H2BGFP, and related either to Paneth or enteroendocrine precursors, or to Hopx/Bmi-positive ‘+4’ cells (Buczacki, 2019; Li et al., 2016; Roth et al., 2012). Paneth, enteroendocrine cell markers, and Hopx and Bmi1 transcripts do not show up in genes differentially expressed in the Vil1Cre-negative cells identified in the present study. This suggests a different origin, in agreement with their Lgr5-independent characteristics. In addition to ribosomal protein genes characteristic of CBCs, the Vil1Cre-negative cluster displays upregulation of nuclear-encoded mitochondrial proteins, including complex IV cytochrome oxidases, mitochondrial ATP synthase subunits and the ATPase inhibiting factor. This may seem unexpected, because quiescent stem cells of diverse origin are reported to rely on glycolysis for energy supply (Shyh-Chang and Ng, 2017). However, contrary to what was expected from earlier measurements, quiescent haematopoietic stem cells have been shown to harbour more mitochondria than their more differentiated counterparts, making them ready to rapidly engage in regeneration when needed (Filippi and Ghaffari, 2019; Morganti et al., 2022).

An unexpected observation of our study is the effect of expression of the diphtheria toxin receptor (HB-Egf) in CBCs, in the absence of toxin administration. The mere expression of HB-Egf significantly increases the number of cells not traced by the Vil1Cre transgene, suggesting the presence of a chronic injury (Chen et al., 2010). This chronic effect is neglected in the numerous studies using the diphtheria toxin receptor to ablate specifically a cell type, which might deserve re-examination.

In conclusion, our results indicate that a hierarchical stem cell model involving a previously unreported lineage of reserve stem cells applies to regeneration of the intestinal epithelium in addition to the plasticity model. Future work will be necessary to assess the relative importance of these two models in various injury situations.

### Limitations of the study

The fact that the newly identified intestinal lineage is only characterized ‘by default’ (absence of the Vil1Cre transgene expression) constitutes clearly a limitation of our study. While it allowed purification of the corresponding cells from FACS-sorted PlusMinus cells, it makes it difficult to identify these cells in the homeostatic wild-type intestinal epithelium and to trace them during development. Future studies exploiting novel lineage-tracing methodologies (Baron and van Oudenaarden, 2019; Jindal et al., 2023; Wagner and Klein, 2020) might allow identification of specific markers of these cells (if such markers exist) that would, in turn, pave the way to their detailed characterization.

## MATERIALS AND METHODS

### Experimental animals

All the animal procedures complied with the guidelines of the European Union and were approved by the local ethics committee of the faculty of Medicine of Université Libre de Bruxelles (protocols 713N and 759N). Mice strains used include: CD1 (Charles Rivers, France), Lgr4/Gpr48^Gt (Leighton et al., 2001), Lgr5-DTR knock-in (Tian et al., 2011), Lgr5-CreERT2 (Jackson Laboratory), Lgr5fl/fl (de Lau et al., 2011), Mcl1fl/fl (Vikstrom et al., 2010), Tg(Vil1Cre)997Gum/J (Madison et al., 2002), Tg (Vil1Cre/ERT2)23Syr (Sylvie Robine, Paris, France), Rosa26YFP, Rosa26LacZ and Rosa26Tomato. Eight- to 16-week-old mice were used for these experiments. The day the vaginal plug was observed was considered as embryonic day 0.5 (E0.5).

### Lineage tracing and specific cell ablation

For lineage-tracing experiments, tamoxifen (Sigma-Aldrich) was dissolved in a sunflower oil (Sigma-Aldrich) and ethanol mixture (19:1) at 10 mg/ml and used in all experiments at a dose of 0.066 mg/g of body weight. For ablation of CBCs, Lgr5-DTR mice were injected intraperitoneally with 50 µg/kg diphtheria toxin (DT; Sigma-Aldrich). Control mice were either injected with sterile Dulbecco's phosphate-buffered saline (DPBS, Gibco) solution or not injected.

### *Ex vivo* culture Matrigel domes (old protocol)

For *ex vivo* organoid culture, the segment of the intestine used for spheroid production is a 5 cm region starting 0.5 cm after the gastric-duodenal junction. This segment was cut into 3-5 mm pieces and incubated in 1- and 5-mM ethylenediaminetetraacetic acid (EDTA) in Dulbecco's phosphate-buffered saline (DPBS) at 4°C (15 min and 25 min, respectively). Epithelial crypts were cultured according to Sato et al. (2009) in advanced-DMEM/F12 medium (Thermo Fisher Scientific) supplemented with 20 mM L-glutamax (Gibco), 1×N2 (Gibco), amphotericin, B27 without vit.A (Gibco), penicillin-streptomycin cocktail, 10 mM N-2-hydroxyethylpiperazine-N-2-ethane sulfonic acid (HEPES), 40 µg/ml gentamycin (all from Thermo Fisher Scientific) and 1 mM N acetyl cysteine (Sigma). Unless indicated otherwise, the medium contained ‘ENR’ [50 ng/ml EGF, 100 ng/ml Noggin (both from Peprotech) and 100 ng/ml CHO-derived R-spondin1 (R&D System)].

Fetal spheroids were prepared and cultured in ENR-containing medium as described previously (Fernandez et al., 2016). Adult spheroids were initially observed when replacing the EDTA step in Sato's protocol by collagenase I (0.132 mg/ml, Sigma-Aldrich) and dispase (0.66 mg/ml, Gibco) treatment of intestinal tissue for 30 min at 37°C. Thereafter, a two-step protocol was devised as follows: the tissue was processed according to Sato et al. (2009), with the EDTA fraction generating organoids; the materials retained on the 70 µm cell strainer (Corning) were washed with Hanks' Balanced Salt Solution (HBSS, Gibco) and centrifuged at 300 ***g*** for 5 min at room temperature. This washing step was repeated two more times. The pellets were treated with 0.132 mg/ml collagenase, 0.66 mg/ml dispase and 0.05 mg/ml DNase1 (Roche Diagnostics) dissolved in Dulbecco‘s Modified Eagle Medium (DMEM, Gibco) for 15 min at 37°C under agitation at 75 rpm. Mixing by up and down pipetting was then performed with a 10 ml pipette before a further 20 min incubation at 37°C. The up and down mixing step was repeated, which resulted in complete dissociation. The dissociated samples were passed through a 100 µm cell strainer (VWR) and centrifuged at 300 ***g*** for 10 min. The pellets were washed twice with basal culture medium [BCM; containing Advanced-DMEM/F12 medium supplemented with 1×penicillin-streptomycin cocktail (100×), 1×amphotericin and 0.8 µl/ml gentamycin] plus 2 mM EDTA (Invitrogen), followed by two washes with DPBS (Gibco) containing 10% fetal bovine serum (FBS). All washes were performed at room temperature and involved centrifugation at 300 ***g*** for 5 min. Finally, the resulting fraction was plated in basement membrane matrix, LDEV-free Matrigel (Corning). The culture medium for the first 48 h was ENR in advanced-DMEM/F12 medium (Thermo Fisher Scientific) supplemented with 10 µM Y-27632 (Peprotech), as described above, in all initial seedings. The medium was changed every other day either with ENR or BCM, as reported in the Results section. When spheroids are cultured in ENR, they must be replated every other day to retain a clear phenotype (otherwise they start shrinking and become dark), whereas when cultured in BCM, they were replated every 4-6 days. Images were acquired with a Moticam Pro camera connected to a Motic AE31 microscope.

Intestinal fibroblasts were isolated and cultured as described previously (Powell et al., 2011) in ENR medium and their supernatant collected after 3 days. This supernatant was used as conditioned medium. In co-culture experiments, organoids were cultured in medium containing 50% fibroblast conditioned medium with 50% organoid culture medium (vol/vol, organoid culture medium was made of advanced-DMEM/F12 medium with 2% FBS and ENR). Regarding the differentiation attempts, Iwp2, DAPT or Verteporfin compounds (all from Sigma-Aldrich) were dissolved in DMSO and added together with fresh ENR medium.

### Sandwich Matrigel protocol

The protocol was derived from Bues et al. (2020). The tissue samples were dissociated as described above, and the final pellets were dissolved in ENR and spread in wells previously coated with solidified Matrigel. After 1 h in the incubator (37°C, 5% CO2) the medium with unattached material was removed. A second layer of Matrigel was added on top of the cells attached to the first layer. After Matrigel solidification, ENR medium supplemented with 10 µM Y-27632 was added to each well. This protocol was used only for initial seeding of adult spheroids.

### Tissue processing and immunohistochemical analysis

The whole small intestine was surgically removed and washed with ice-cold Ca^2+^- and Mg^2+^-free DPBS. The intestine was cut open longitudinally with the mucosa facing outwards and was rolled around a 1 ml pipette from the duodenum toward the ileum. The tissue samples were immediately fixed with a 10% formalin solution (VWR), overnight at room temperature, and then incubated in succession in a 20% and a 30% sucrose solution for at least 24 h each before being embedded in tissue freezing medium (Leica). Histological, immuno-fluorescence and histochemistry experiments were carried out on 6 µm sections. A 10 mM sodium citrate solution (pH 6) was used as an epitope retrieval solution when required. The tissue samples were incubated with primary antibodies overnight at 4°C. The secondary anti-species biotin- or fluorochrome-coupled antibodies were incubated for a minimum of 1 h at room temperature. An ABC kit (Vector Labs) and a DAB substrate Kit (Vector Labs) were used for target revelation in immunohistochemistry. Haematoxylin (immunohistochemistry) or DAPI (immunofluorescence) were used for nuclei staining, according to standard procedures. The primary and secondary antibodies used for staining are listed in Table S4. After dehydration, slides were mounted in a xylene-based medium (Coverquick 4000, VWR Chemicals) in the case of immunohistochemistry or with glycerol mounting medium (Dako) containing 2.5% 1,4 Diazabicyclo [2.2.2] octane (Sigma-Aldrich) in the case of immunofluorescence. A Nanozoomer digital scanner (Hamamatsu), and a Zeiss Axio Observer inverted microscope and Aurox spinning disk (immunofluorescence) were used for visualization of the samples. The number of animals used for each experiment is reported in the figures or figure legends.

Regarding *ex vivo* culture, two fields for imaging were chosen at the start of the experiment for each animal and pictures were taken at different time points. Settings for organoids and spheroids were always the same (Fig. 4A-D,G,H). X-gal staining was performed for 5 h on organoids and spheroids at day6 (Fig. 4B).

### RNA seq and gene set enrichment analysis

RNA was extracted using mirVana miRNA Isolation Kit (Invitrogen). A total of 400 ng extracted RNA was quality controlled using a Fragment Analyzer 5200 (Agilent Technologies). Indexed cDNA libraries were obtained using the TruSeq stranded mRNA LP (Illumina) following the manufacturer's recommendations. The multiplexed libraries were loaded on a NovaSeq 6000 (Illumina) and sequences were produced using a 200 Cycles Kit. Approximately 25 million paired-ends reads were mapped against the mouse reference genome GRCm38 using STAR software to generate read alignments for each sample. Annotations Mus-musculus.GRCm38.90.gtf were acquired from ftp.Ensembl.org. After transcript assembling, gene-level counts were obtained using HTSeq. Genes differentially expressed in adult spheroids (as compared with organoids or fetal spheroids) were identified using the EdgeR method [minimum 10 counts per million (CPM), FDR<0.05] and further analysed using GSEA MolSig (Broad Institute) (Subramanian et al., 2005) to identify biological processes.

### ATAC-seq experiment

Adult spheroids and organoids were cultured as described previously. 41,000 sorted cells were harvested in 1 ml of PBS+3% FBS at 4°C. After centrifugation, cell pellets were resuspended in 100 ml of lysis buffer (Tris HCl 10 mM, NaCl 10 mM, MgCl_2_ 3 mM and Igepal 0.1%) and centrifuged at 500 ***g*** for 25 min at 4°C with the break set at 4. The supernatant was discarded, and nuclei were resuspended in 50 ml of reaction buffer [TDE1 transposase 2.5 ml (Illumina) and TDE buffer 25 ml (Illumina)]. The reaction proceeded for 30 min at 37°C and was then terminated by the addition of 5 ml of stop buffer (NaCl 900 mM, EDTA 300 mM). DNA purification was performed using the MinElute purification kit (QIAGEN) according to the manufacturer's instructions. DNA libraries underwent PCR amplification (NEB-Next High-Fidelity 2× PCR Master Mix, New England Biolabs), indexing using previously described primers (Buenrostro et al., 2015) and double-size selection from 150 to 1200 base pairs (bp) using AmpureXP magnetic beads (Beckman) as per the manufacturer's protocol. The multiplexed libraries were loaded onto a NovaSeq 6000 (Illumina) using a S2 flow cell, and paired-end sequences were generated using a 200 Cycle Kit. ATAC-seq paired-end reads were subsequently aligned to the mouse GRCm38 genome using Bowtie2 (version 2.2.6) with specified options. A list of ATAC-seq oligos for PCR are listed in Table S4.

### Gene expression analysis by qPCR and RNAscope

qRT-PCR was performed on total RNA extracted from adult spheroid or organoid cultures using the mirVana miRNA Isolation Kit (Invitrogen) according to manufacturer's recommendations. A DNase I treatment (Invitrogen) was used to remove potential contaminant genomic DNA. cDNA was prepared using RnaseOUT and Superscript II according to manufacturer's manual (Invitrogen). qTower 3 from Analytik Jena was used to perform qPCR. Gene expression levels were normalized to that of reference genes (*Rpl13*, *Sdha* and *Ywhaz*) and quantified using the qBase Software (Biogazelle). Primer sequences are reported provided in Table S4. *In situ* hybridization experiments were performed based on the instructions provide with the RNAscope kit (ACD-Biotechne; probes are listed in Table S4).

### Flow cytometric analysis, cell sorting and single cell RNAseq

After removal of the EDTA fraction, samples from 5 Vil1Cre (heterozygotes, HE), Rosa26R-Tomato (HE) mice were treated with collagenase (0.132 mg/ml), dispase (0.66 mg/ml) and DNase1 (0.05 mg/ml) solution for 35 min at 37°C, washed twice with BCM-2 mM EDTA solution with centrifugation at 500 ***g*** for 2 min in between. The cell pellet was washed once with PBS-2% FBS (FACS solution) and centrifuged at 500 ***g*** for 2 min. The cells were then incubated with rat anti-mouse CD16/CD32 (blocking solution) at a final dilution of 1/50 (v:v) in FACS solution for 15 min. The APC rat anti-mouse CD31 (1/200), APC rat anti-mouse CD45 (1/500) and BV711 rat anti-mouse CD326 (Epcam, 1/200) antibodies were added to blocking solution and incubated for 45 min on ice. After three washes with FACS solution, DAPI was added to mark the dead cells (1/2000) and the cells were sorted using the FACS Aria I cytometer (BD Biosciences). 20,000 Epcam-positive and Tomato-negative (PlusMinus) or Epcam-positive and Tomato-positive (PlusPlus) cells were collected for each animal. These cells were processed through the Chromium Next GEM Single Cell 3′ Reagent Kits v3.1 (10X Genomics) according to manufacturer's recommendations and sequenced on a Novaseq6000 (Illumina). The data were processed through the CellBender software to decrease contamination by ambient RNA (Fleming et al., 2023). Data were analysed using the Seurat Package in R (Stuart et al., 2019). For PlusMinus sample, 2650 cells passed the quality control steps (250-2000 counts and <10% of mitochondrial genes). For PlusPlus sample, 2154 passed the quality control steps (250-8000 features and <10% of mitochondrial genes). SCTransform was used as the normalization and scaling method (Hafemeister and Satija, 2019). The UMAP plots were generated using 15 dimensions and a clustering resolution of 0.4 for the PlusMinus sample, 15 dimensions and a clustering resolution of 0.6 for the PlusPlus sample, and 15 dimensions and a clustering of 0.3 for the PlusMinus/PlusPlus merged analysis. Cell types were identified based on previous scRNAseq experiments (Haber et al., 2017) and GSEA MolSig (Broad Institute) (Subramanian et al., 2005).

### Statistical analyses

Statistical analyses were performed with Graph Pad Prism 9. Venn diagrams in and Figs. S2, S4 and S7 were created using Venny, version 2.0.2 (https://bioinfogp.cnb.csic.es/tools/venny/) and analysed with the hypergeometric p-value calculator (https://systems.crump.ucla.edu/lab-software/). All experimental data are expressed as mean±s.e.m. The significance of differences between groups was determined by appropriate parametric or non-parametric tests, as described in figure legends.

## Supplementary Material



10.1242/develop.204654_sup1 Supplementary information

Table S1. Bulk RNAseq comparing transcriptomes of organoids and adult spheroids cultured in different conditions: ENR, BCM short term (passage 6, day 6), BCM long term (passage 26, day 6).

Table S2. Bulk RNAseq comparing transcriptomes of adult and fetal spheroids.
