# Supplementary material for: A Lgr5-independent developmental lineage is involved in mouse intestinal regeneration
Source: Development. 2025 Apr 10;152(7):dev204654. doi: 10.1242/dev.204654 (PMC12045596; doi:10.1242/dev.204654)
Supplement: Supplementary information [file develop-152-204654-s1.pdf]

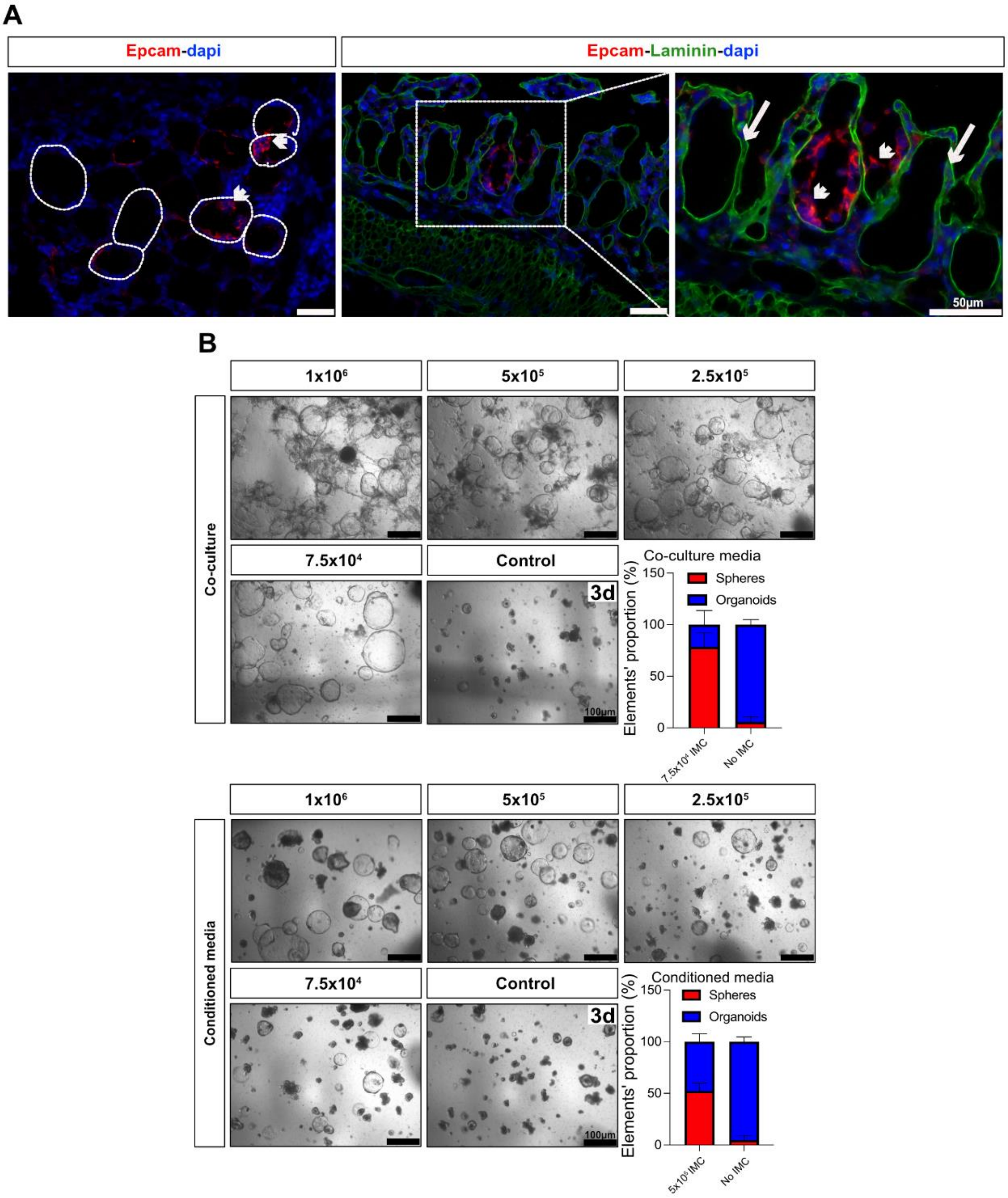

Fig. S1. Adult spheroids in ex vivo culture. (A) Immunofluorescence showing section of the material remaining on the filter after EDTA treatment of the intestinal tissue (see **Fig.1B** and Methods); round empty structures correspond to crypt ghosts (dotted contour) surrounded by basal membrane (Laminin) and mesenchyme (arrows), remaining epithelial cells are Epcam positive (arrow heads). (B) Panel showing culture of EDTA-derived organoids in the presence of different numbers of intestinal mesenchymal cells (IMC) in co-culture (upper panel) or in the presence of conditioned media derived from IMC plated at different densities (lower panel); graphs show the proportion of spheroids/organoids elements observed at day3 of culture using 7.5x10^4 and 5x10^5 IMC as an example (n=2 different organoids samples for co-culture, n=3 different organoid samples for conditioned media). Scale bars: 50 µm for IF and 100 µm for bright field.

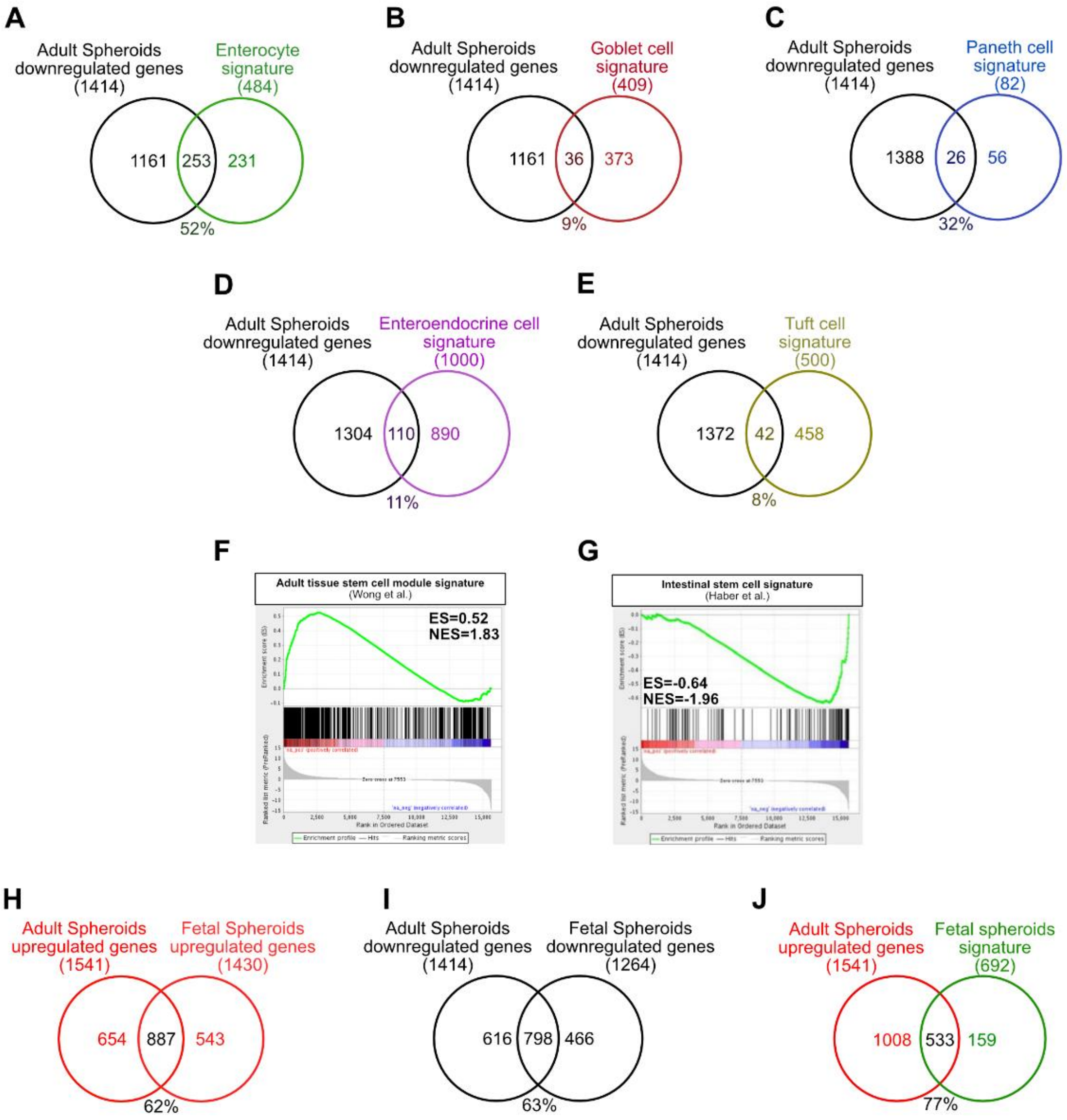

Fig. S2. Adult spheroids are made of poorly differentiated intestinal epithelial cells sharing similarities with fetal spheroids.(A-E)Venn diagrams showing the proportion of genes downregulated more than 2-fold in adult spheroids (compared to organoids) that belong to the various differentiated intestinal cell type signatures (Haber *et al.*, 2017). Percentages show genes belonging to each signature that are downregulated. (F) Positive correlation of the same set of genes with the adult stem cell module described by Wong et al. (Wong *et al.*, 2008) and (G) pre-ranked GSEA analysis showing negative correlation between adult spheroid up-regulated genes (versus organoids) and the intestinal stem cell signature described by Haber et al. (Haber *et al.*, 2017). ES = enrichment score, NES = normalized enrichment score. Venn diagrams showing the common number of genes, similarly (H) up- or (I) down-regulated more than 2-fold (when compared to organoids), between adult or fetal intestinal spheroids. (J) Venn diagram showing the number of genes more than 2-fold upregulated in adult intestinal spheroids (when compared to organoids) that belong to the fetal intestinal/stomach spheroid signature previously described (Fernandez *et al.*, 2016).

Development • Supplementary information

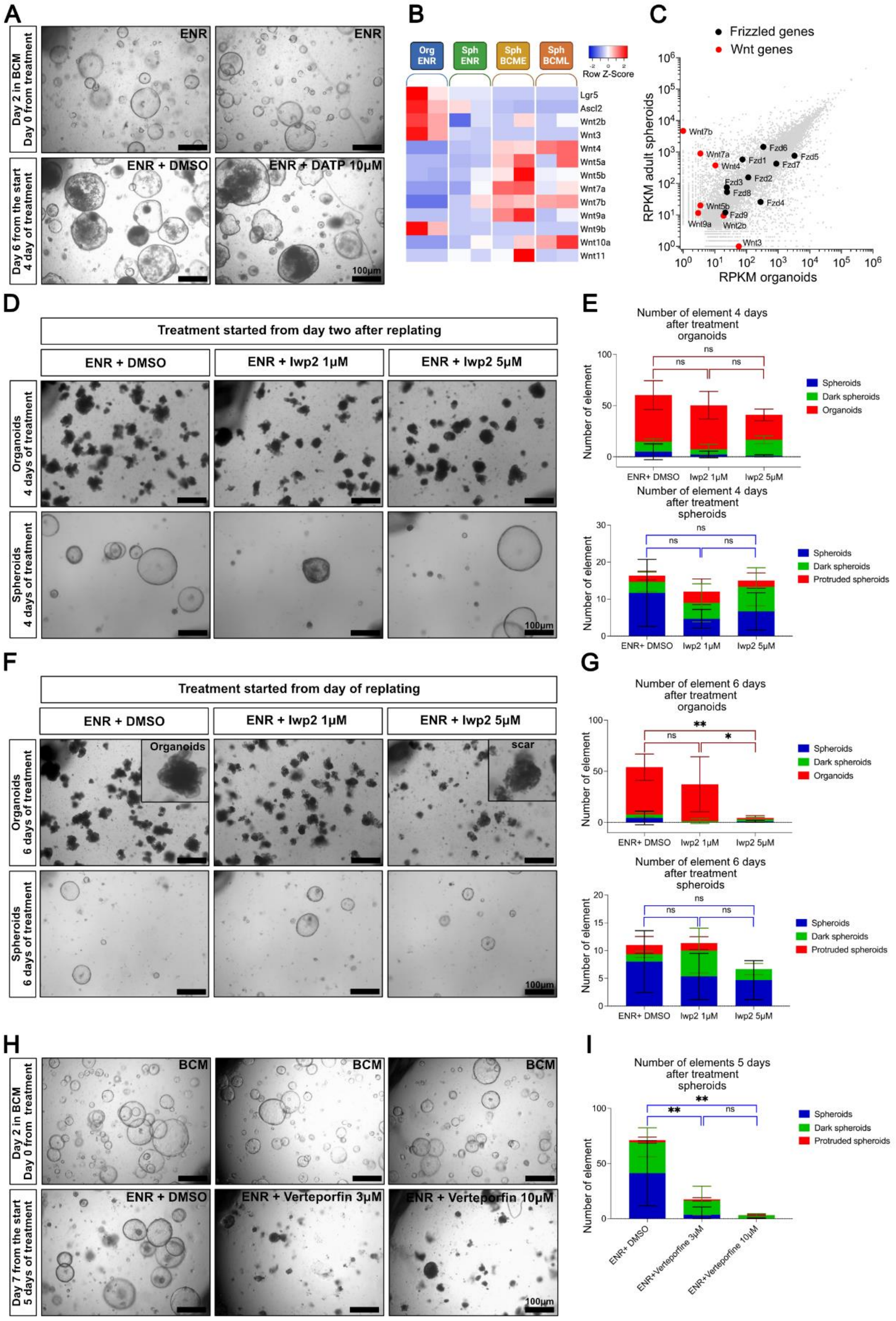

Fig. S3. The phenotype of adult spheroids is extremely stable. Attempts were made to differentiate adult spheroids stably cultured in BCM into organoids by transferring them to ENR containing a series of agents known to affect intestinal differentiation: Aspirin, cAMP agonists/antagonists, YapTaz inhibitors (verteporfin and CA3), valproic acid, Notch inhibitors (DAPT, DBZ, LY511455), all-trans retinoic acid, NFkB inhibitors (TCPA, BMS), TGFbeta

Development • Supplementary information

inhibitor (SB431542), porcupine inhibitor (Iwp2). None proved effective. Illustrated in the figure are: (A) the absence of effects of DAPT on spheroid morphology (representative field of culture from 4 animals); (B) Heatmap documenting relative expression of Wnt genes in organoids and spheroids (n=2); (C) Logarithmic plot showing level of Wnt (red) and Frizzled (black) gene expression in adult spheroids and organoids (for details see Table S1); (D-G) effects of Iwp2 treatment for 4 days, starting 2 days after replating of organoids or spheroids in 1 or 5 µM of Iwp2 (D,E, n=3), or for 6 days, immediately following replating from BCM (F,G, n=3); effects of Verteporfin after 5 days of exposure to 5 or 10 µM of the drug, starting 2 days following replating from BCM (H,I, n=4). All agents were dissolved in DMSO, 0.5% in control and experimental samples. Quantifications (panels E,G,I) were performed from analyses of random fields selected at the initiation of the treatments (Mean ± standard error of the mean). Statistical analyses: two-way ANOVA test with Tukey's multiple comparison to (panel E, organoids (upper)/spheroids (lower)); (panel G, organoids(upper)/spheroids(lower)); (panel I, spheroids): ns $p>0.05$; *$p<0.02$; **$p<0.01$.

Development • Supplementary information

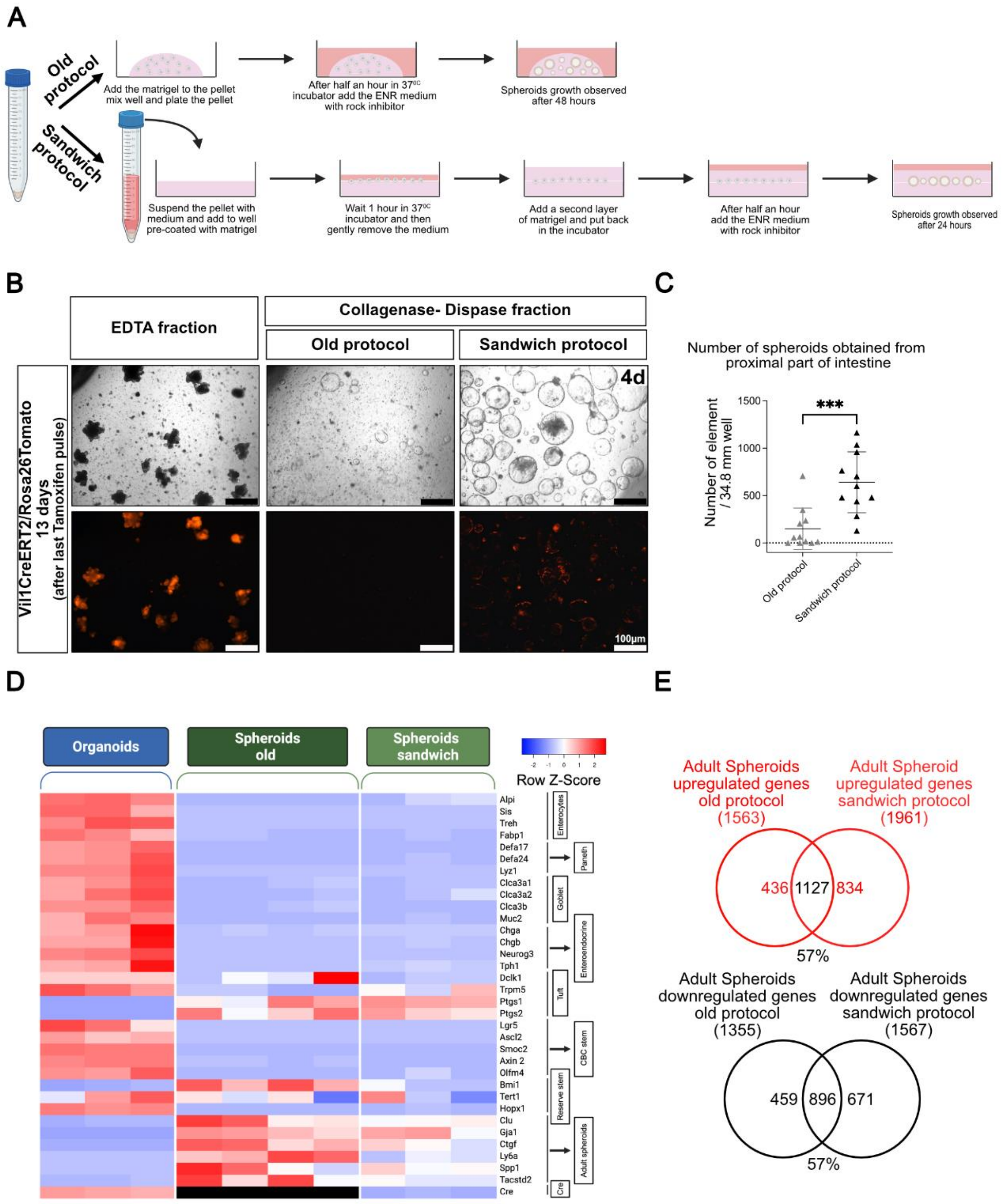

Fig. S4. Comparison of adult spheroids obtained with two experimental protocols. (A) Outline of the two protocols. (B) Representative pictures of organoids (EDTA fraction) and spheroids (old or sandwich protocols) of collagenase/dispase fraction obtained from Vil1CreERT2/Rosa26Tomato mice after 3 pulses of Tamoxifen. The animals were sacrificed at day 16 and the pictures were taken at day 4 of culture. (C) Comparison of the yield of spheroids obtained with the two protocols. Each symbol illustrates results from one mouse. (D) Heatmap from bulk RNA sequencing showing similarity between expression of differentiation-, CBC- and spheroid-specific genes in spheroids obtained with the two protocols. Note that Cre transcripts were only assayed in the “new protocol”. (E) Venn diagrams showing the number of genes, similarly up- or downregulated more than 2-fold when compared to organoids. Statistical analyses: unpaired t test: *** $p= 0.0005$.

Development • Supplementary information

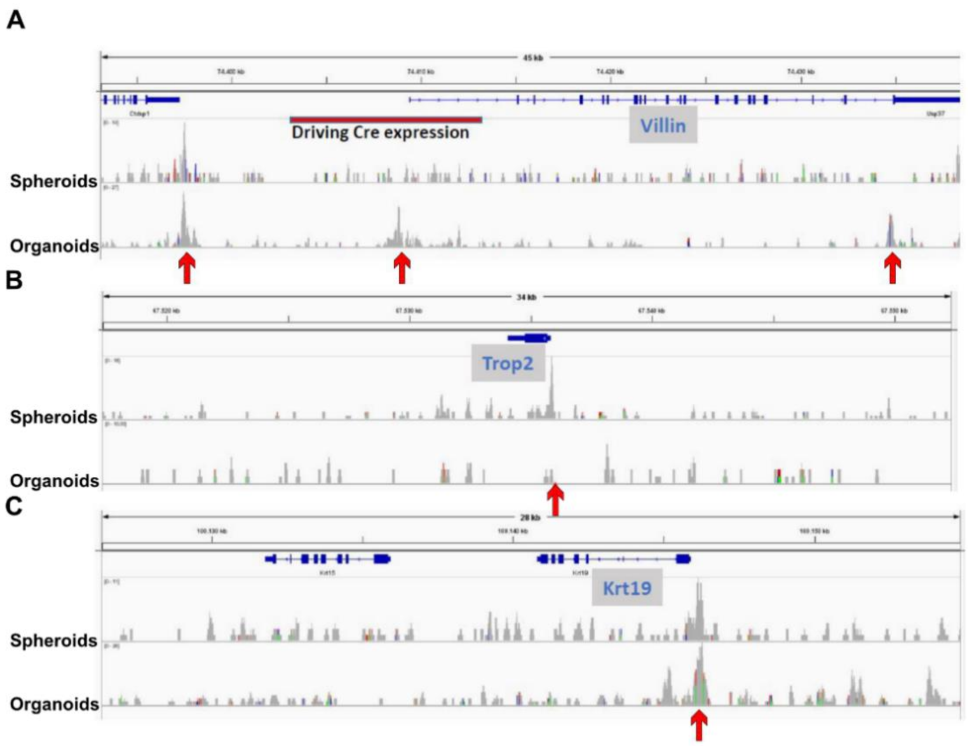

Fig. S5. Different organization of chromatin around the villin gene of adult spheroids and organoids. (A) AtacSeq experiment displaying DNA accessibility in the vicinity of the endogenous villin gene from spheroids and organoids in chromatin from a Vil1Cre/ Rosa26Tomato mouse. (B) Results centered on Trop2 gene and (C) Krt19 gene illustrates control genes, expressed specifically in adult spheroids, or in both spheroids and organoids, respectively. Arrows point to the observed differences.

Development • Supplementary information

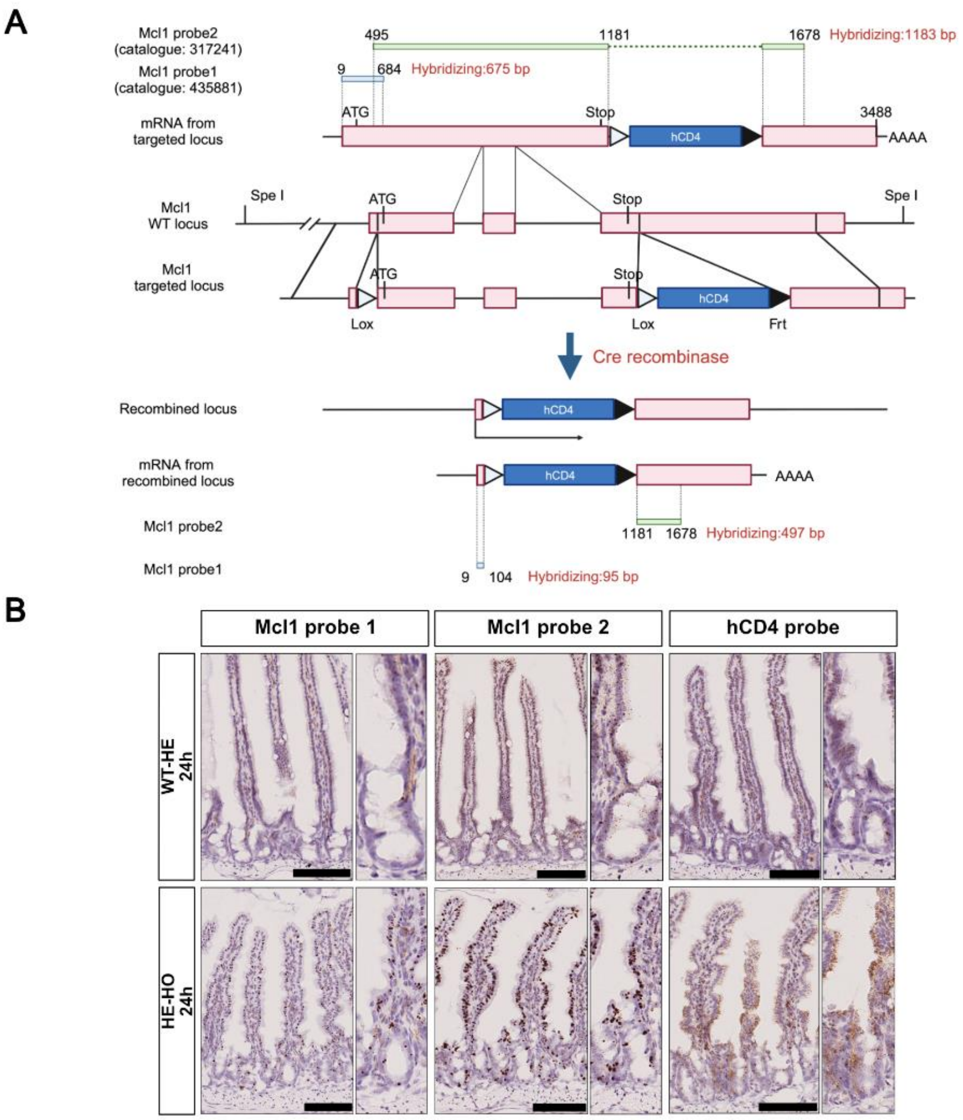

Fig. S6. In situ hybridization (ISH) of two Mcl1 probes to mRNA transcribed from non-recombined and recombined Mcl1 loci from Mcl1-floxed mice. (A) Schematic representation of the non-recombined and recombined locus, with the corresponding mRNAs and indication of the extent of the Mcl1 probes. (B) Representative pictures of ISH with the two Mcl1 probes and the hCD4 probe to intestinal sections of Mcl1$^{fl/wt}$ or VilCreERT2/Mcl1$^{fl/fl}$ mice 24 hours after tamoxifen administration, showing the paradoxical increase of the signals.

Development • Supplementary information

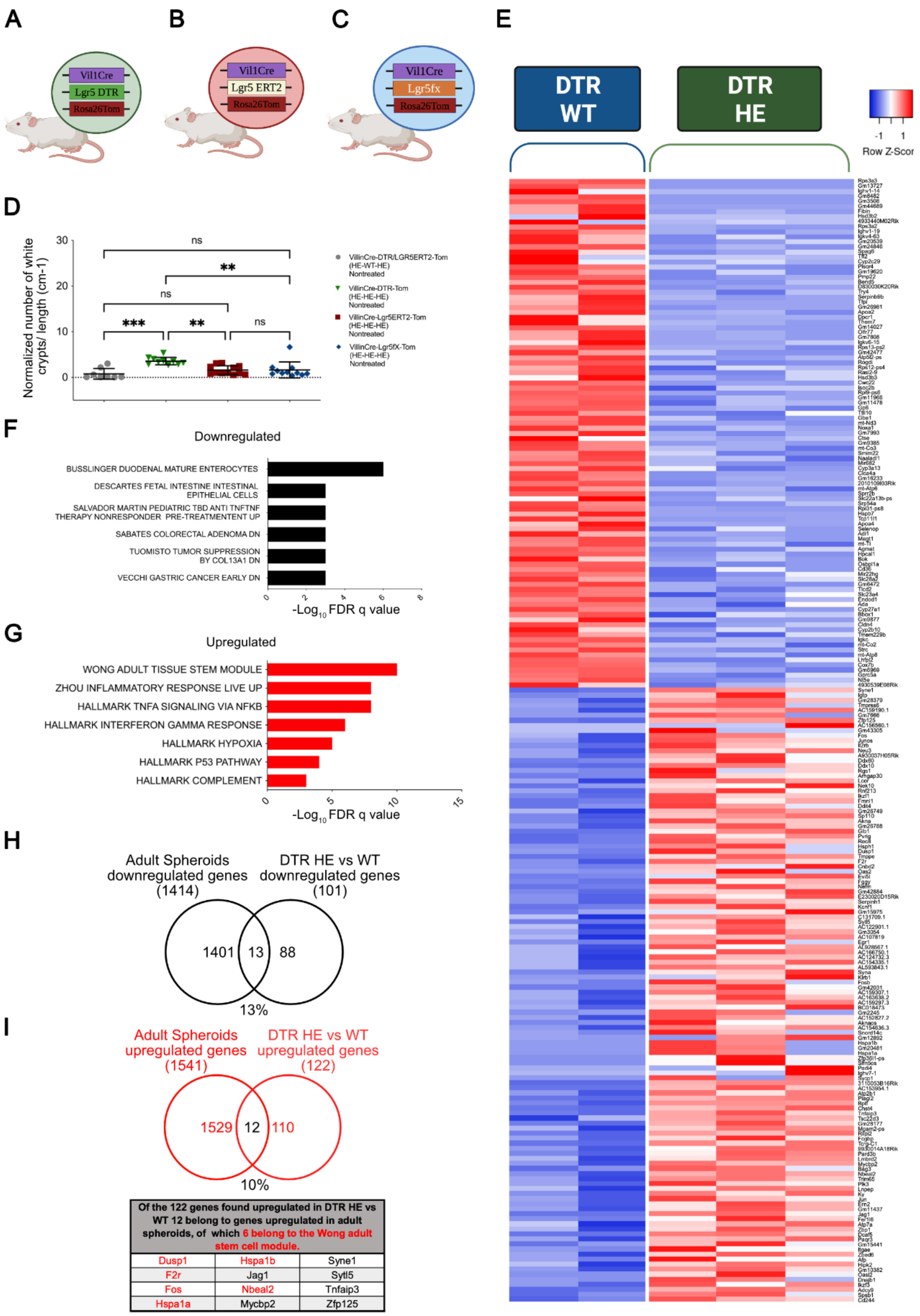

Of the 122 genes found upregulated in DTR HE vs WT 12 belong to genes upregulated in adult spheroids, of which 6 belong to the Wong adult stem cell module.		
Dusp1	Hspa1b	Syne1
F2r	Jag1	Sytl5
Fos	Nbeal2	Tnfaip3
Hspa1a	Mycbp2	Zfp125

Fig. S7. Expression of the diphtheria toxin receptor triggers a regeneration-like phenotype in the intestinal epithelium in the absence of diphtheria toxin. The three transgenic mouse lines used in these experiments were on a Vil1Cre/Rosa26Tomato background. All animals were untreated and sacrificed when they were 2 months old. (A) Lgr5-DTR, (B) Lgr5-CreERT2, (C) Lgr5 with a floxed exon 16 allowing its deletion in the Vil1Cre background. (D) Quantification of un-recombined (white) crypts in Vil1Cre/

Development • Supplementary information

Rosa26Tomato double heterozygotes, Vil1Cre/Lgr5DTR/Rosa26Tomato, Vil1Cre/Lgr5-CreERT2/ Rosa26Tomato and VilCre/Lgr5$^{fl/wt}$/Rosa26Tomato triple heterozygotes. All mice except VilCre/Rosa26Tomato had one Lgr5 allele deleted or non-functional. (E-I) Bulk RNAseq analyses of RNA extracted from uncultured total EDTA-released material; (E) Heatmap from bulk RNAseq of Vil1Cre/Rosa26Tomato double heterozygotes (DTR WT) compared to Vil1Cre/Lgr5DTR/Rosa26Tomato triple heterozygotes (DTR HE). GSEA biological processes of (F) downregulated (C2, C8 cell signature) and (G) upregulated genes (C2 and hallmark genes) were shown in the transcriptome of Lgr5-DTR heterozygous versus wild type crypts. Venn diagrams showing common downregulated (H) or upregulated (I) genes in adult spheroids (versus organoids) and DTR-expressing (versus wild type) intestine. Statistical analyses: One-way ANOVA test with Tukey's multiple comparison tests: ns $p>0.05$; *** $p=0.0002$.

Development • Supplementary information

Table S1. Bulk RNAseq comparing transcriptomes of organoids and adult spheroids cultured in different conditions: ENR, BCM short term (passage 6, day 6), BCM long term (passage 26, day 6).

Available for download at
https://journals.biologists.com/dev/article-lookup/doi/10.1242/dev.204654#supplementary-data

Table S2. Bulk RNAseq comparing transcriptomes of adult and fetal spheroids.

Available for download at
https://journals.biologists.com/dev/article-lookup/doi/10.1242/dev.204654#supplementary-data

Table S3. GSEA MolSig analyses for hallmarks characterizing the adult spheroid transcriptome compared to fetal spheroids.

Hallmark – Description	*p-value*	FDR *q-value*
Genes involved in epithelial to mesenchymal transition	2.46 e^{-53}	1.23 e^{-51}
Genes regulated by NF-κβ in response to TNFα	2.45 e^{-49}	6.12 e^{-48}
Genes up-regulated by KRAS activation	4.85 e^{-32}	8.09 e^{-31}
Genes defining inflammatory response	9.12 e^{-30}	1.14 e^{-28}
Genes encoding components of blood coagulation system	8.17 e^{-27}	8.17 e^{-26}
Genes up-regulated in response to hypoxia	1.86 e^{-26}	1.33 e^{-25}
Genes up-regulated in response to IFNγ	1.86 e^{-26}	1.33 e^{-25}
Genes up-regulated in response to IFNα proteins	4.03 e^{-23}	2.52 e^{-22}

Table S4. Origin of animals and reagents; list of primers.

Transgenic mouse lines	
B6.Cg-Tg(Vil1-cre)997Gum/J (referred as VillinCre)	Jax mice/ Strain #004586
CD1	Charles Rivers, France
Lgr4/Gpr48^Gt mice	Leighton, 2001 6712 /id
Lgr5-CreERT2	Jackson Laboratory
Lgr5-DTR	Tian et al.
Lgr5fl/fl	De Lau et al
Mcl1fl/fl	Vikstrom, 2010 7392 /id
Rosa26LacZ	Jackson Laboratory
Rosa26Tomato	Jackson Laboratory
Rosa26YFP	Jackson Laboratory
Tg (Vil-cre/ERT2)23Syr (referred to as VilCreERT2)	Sylvie Robine, Paris, France

Transgenic mouse lines	Genotyping primers			
	Primer 1	Primer 2	Primer 3	Primer 4
VillinCre	GTGTGGGACAGAAC AAACC	ACATCTTCAGGTT CTGCGGG	-	-
Lgr4/Gpr48^Gt mice	CCAGTCACCACTCTT ACACAATGGCTAAC	ATTCCCGTAGGAG ATAGCGTCCTAG	GGTCTTTGAGCA CCAGAGGAC	-
Lgr5-CreERT2	CTGCTCTCTGCTCCC AGTCT	ATACCCCATCCCTT TTGAGC	GAACTTCAGGGT CAGCTTGC	-
Lgr5-DTR	CGACAACCACTACCT GAGCA	CGGGACCAGATGC GATA	AGCTAGGCTCTG CTCTGTCA	-
Lgr5fl/fl	CACCATTGTGCAAAT AGC	GATTATAGAAATG CCATCTGC	CTTGAACTTGAA ATAACCCAGC	-
Mcl1fl/fl	GCACAATCCGTCCGC GAGCCAA	GCCGCAGTACAGG TTCAAG	-	-
Rosa26LacZ	AAAGTGGCTCTGAGT TGTTAT	GCCAAGAGTTTGT CCTCAACC	GGAGCGGGAGA AATGGATATG	-

Development • Supplementary information

Development: doi:10.1242/dev.204654: Supplementary information

Rosa26Tomato	GGCATTAAAGCAGC GTATCC	AAGGGAGCTGCAG TGGAGTA	CCGAAAAATCTG TGGGAAGTC	CTGTTCCTGTACG GCATGG
Rosa26YFP	AAAGTCGCTCTGAGT TGTTAT	GGAGCGGGAGAA ATGGATATG	AAGACCGCGAA GAGTTTGTC	-
VilCreERT2	CAAGCCTGGCTCGAC GGCC	GCACAATCCGTCC GCGAGCCAA	-	-

Antibodies	Company	Catalogue Number	Dilution
APC rat anti-mouse CD31	BD Pharmingen™	551262	1/200
APC rat anti-mouse CD45	BD Pharmingen™	561018	1/500
BV711 rat anti-mouse CD326	BD Horizon™	563134	1/200
Donkey anti-mouse-Cy3	Jackson ImmunoResearch	711165150	1/500
Donkey anti-rabbit-AF488	Jackson ImmunoResearch	711485152	1/500
Donkey anti-rabbit-biotinylated	Jackson ImmunoResearch	711065152	1/500
Donkey anti-rat-biotinylated	Jackson ImmunoResearch	712065150	1/500
Goat anti-Tdtomato	Sicgenantibody	AB8181	1/100
Mouse anti-Cdh17	R&D systems	MAB1032	1/100
Mouse anti-Cdx2	BioGenex	CDX2-88	1/100
Mouse anti-Laminin	Sigma Bio Science	L8271	1/2000
Purified rat anti-mouse CD16/CD32 (mouse BD FC block™)	BD Pharmingen™	553142	1/50
Rabbit anti-Epcam	Abcam	AB71916	1/250
Rabbit anti-RFP	Rockland	600-401-379	1/250
Rabbit anti-Villin	Invitrogen	PA5-220072	1/500
Rabbit cleaved Caspase-3	Cell Signalling Technology	9661	1/300
Rat 5f8 Red Fluorescent Proteins (RFP)	Chromotek	5F8	1/1000

qPCR primers	Primer forward	Primer reverse
Cre	GTGGTCTGGCAGTAAAAACTATC	GTGAAACAGCATTGCTGTCACTT
mouse Rpl13	CCCGTGGCGATTGTGAA	TCATTGTCCTTCTGTGCAGGTT
mouse Sdha	CCTTCACGGATCTCAAGAGTCAT	AGTCAAGGTGAAAGGTTCATGGA
mouse Villin	TTCTCTGGCACCGTCACTC	CGTAGCAAACCCATGTTCCT

ATAC-seq oligos for PCR	Sequence
Ad1_noMX	AATGATACGGCGACCACCGAGATCTACACTCGTCGGCAGCGTCAGATGTG
Ad2.15_TGGATCTG	CAAGCAGAAGACGGCATACGAGATCAGATCCAGTCTCGTGGGCTCGGAGATGT
Ad2.16_CCGTTTGT	CAAGCAGAAGACGGCATACGAGATACAAACGGGTCTCGTGGGCTCGGAGATGT
Ad2.17_TGCTGGGT	CAAGCAGAAGACGGCATACGAGATACCCAGCAGTCTCGTGGGCTCGGAGATGT
Ad2.18_GAGGGGTT	CAAGCAGAAGACGGCATACGAGATAACCCCTCGTCTCGTGGGCTCGGAGATGT
Ad2.19_AGGTTGGG	CAAGCAGAAGACGGCATACGAGATCCCAACCTGTCTCGTGGGCTCGGAGATGT
Ad2.20_GTGTGGTG	CAAGCAGAAGACGGCATACGAGATCACCACACGTCTCGTGGGCTCGGAGATGT

Rnascope probes	Company	Catalogue Number

Development • Supplementary information

human CD4	ACD-Biotechne	605601
mouse Clu	ACD-Biotechne	427891
mouse Mcl1 probe 1	ACD-Biotechne	435881
mouse Mcl1 probe 2	ACD-Biotechne	317241

Chemicals, enzymes and other reagents and kits	Company	Catalogue Number
1,4 Diazabicyclo [2.2.2] octane	Sigma-Aldrich	D27802
10% formalin solution, buffered	VWR	11699408
ABC kit	Vector laboratories	PK-4000
Citrate sodium	AnalaR Normapur	27833294
Coverquick 4000	VWR Chemicals	5547539
DAB substrate kit	Vector laboratories	SK-4100
Diphtheria Toxin from Corynebacterium diphtheriae	Sigma-Aldrich	D0564
DNAse I	Invitrogen/	18068015
Ethanol absolute	VWR	20821-296DP
EvaGreen® Dye, 20X in Water	Biotium Glowing Products for Science™	31000
Glycergel Mounting Medium	Dako	C0563
Mayers' hemalun solution	Millipore	1092492500
MinElute PCR Purification Kit	Qiagen	28004
MirVana™ miRNA Isolation Kit	Invitrogen	AM1561
NEBNext® High-Fidelity 2X PCR Master Mix	New England Biolabs	M0541S
Nextera DNA Library Prep kit	Illumina	15028212
RNAscope kit	ACD-Biotechne	322300
RNAse OUT	Invitrogen	10777019
Sucrose	Millipore	1076511000
Sunflower seed oil from Helianthus annuus	Sigma-Aldrich	S5007
Superscript II	Invitrogen	180064014
Tamoxifen	Sigma-Aldrich	T5648
Tissue freezing medium	Leica	14020108926

Development • Supplementary information

Cell culture reagents	Company	Catalogue Number	Final Concentration
100 µm cell strainer	Avantor promoted by VWR	7322759	-
40 µm cell strainer	Avantor promoted by VWR	7322757	-
70 µm cell strainer	Avantor promoted by VWR	7322758	-
Advanced-DMEM/F12 medium	Thermo fisher scientific	12634028	-
Amphotericin	Thermo fisher scientific	152900026	1x
B27 w/o vit.A	Thermo fisher scientific	12587010	1x
Basement membrane matrix, LDEV free Matrigel	Corning	354234	100%
Collagenase I	Sigma Aldrich	C9407	0.132mg/ml
DATP	Sigma-Aldrich	D5942	10µM
Dispase	Gibco	17105041	0.66mg/ml
DMEM 1X	Gibcco	41965039	-
DNase1	Roche Diagnostics Gmbh	10104159001	0.05mg/ml
DPBS 1X	Thermo fisher scientific	14190094	1x
Fetal bovine serum (FBS)	ThermoFisher	10270106	10%
Gentamycin	Thermo fisher scientific	152900026	40 µg/ml
HBSS with CaCl2 and MgCl2	Gibco	14025050	-
HEPES	Thermo fisher scientific	15630080	10 mM
Iwp2	Sigma-Aldrich	I0536	1µM/5µM
L-Glutamax	Gibcco	35050061	20 mM
mouse EGF	Peprotech	315-09	50 ng/ml
mouse Noggin	Peprotech	250-38	100 ng/ml
mouse Rspondin 1	R&D systems	7150-RS-250	100 ng/ml
N-acetyl cysteine	Sigma Aldrich	A9165	1 mM
N2	Thermo fischer scientific	17502048	1x
penicillin-streptomycin cocktail 100X	Thermo fisher scientific	15070063	1x
Ultra-pure EDTA 0.5M, PH 8	Invitrogen	15575038	1mM and 5mM
Verteporfin	Sigma-Aldrich	SML0534	3µM/10µM
Y-27632	Peprotech	1293823	10 µM

Development • Supplementary information
